# Immunoengineering in the field of tendon and bone regeneration: immunomodulatory biomaterials, delivery platforms, and preclinical models for chronic diseases

**DOI:** 10.3389/fbioe.2026.1844904

**Published:** 2026-05-21

**Authors:** Wanxue Wang, Pengfei Yan, Junmiao Liu, Dexi Cui, Qinge Guo, Yu Jiang, Wei Sheng, Yusen Qiao, Xia Zhao

**Affiliations:** 1 Department of Sports Medicine, the Affiliated Hospital of Qingdao University, Qingdao, China; 2 Department of Epidemiology and Health Statistics, Public Health College, Qingdao University, Qingdao, China; 3 Knee Preservation Center, the Affiliated Hospital of Qingdao University, Qingdao, China; 4 Huangshi Love & Health Hospital Affiliated of Hubei Polytechnic University, Huangshi, China; 5 Department of Orthopedics, The First Affiliated Hospital of Soochow University, Suzhou, China

**Keywords:** bone immunology, organoids and organ-on-a-chip, spatial transcriptomics, targeted senescence clearance, tendon-bone interface

## Abstract

The functional and structural reconstruction of the tendon-bone interface (TBI) is a major challenge in orthopedics and sports medicine. Under the influence of chronic degenerative pathologies such as aging, diabetes, and rheumatoid arthritis, the cascading collapse of the local immune-metabolic network disrupts the regenerative microenvironment of the tissue, making the clinical translation of traditional inert physical scaffolds extremely difficult. This review systematically summarizes the latest paradigm shifts in “bone immunoengineering” aimed at overcoming the complex challenges of TBI regeneration. We first decode the core regulatory networks that control interface heterogeneity remodeling, thoroughly analyzing the spatiotemporal polarization dynamics of macrophages, the double-edged effects of the Piezo1-YAP mechanotransduction axis, and the “neuro-immune-skeletal” ternary communication mechanism. Based on this pathological framework, we comprehensively overview next-generation intelligent biophysical and chemical intervention strategies for actively reprogramming extreme microenvironments. These strategies include piezoelectric nanohydrogels for electromechanical-metabolic coupling, Janus asymmetric microfluidic interfaces for multi-ion spatiotemporal rectification, and precise spatial delivery platforms for targeted clearance of senescent cells and engineered exosomes. Furthermore, to overcome the translational barriers between underlying mechanisms and clinical applications, we focus on the cross-scale evolution of preclinical evaluation systems, elaborating on the core value of three-dimensional tendon-bone organoids, microfluidic organ-on-chip systems, and high-resolution spatial transcriptomics. Finally, this review envisions advanced microphysiological systems characterized by closed-loop dynamic adaptive biomaterials, spatiotemporal matching of degradation kinetics, and deep integration with artificial intelligence (AI), highlighting their broad prospects in driving the next-generation of personalized, precise regenerative medicine in orthopedics.

## 1 Introduction

The tendon-bone interface (TBI), also known as the insertion site, is a highly specialised heterogeneous anatomical structure. It facilitates seamless mechanical transmission and stress dissipation between soft and hard tissues through a smooth gradient transition from flexible, parallel tendon collagen fibres, non-calcified fibrocartilage, and calcified fibrocartilage to rigid subchondral bone. However, following acute trauma, the limited endogenous healing capacity of this intricate tissue structure often results in the interface being replaced by disorganised fibrous scar tissue lacking biomechanical strength, making it highly susceptible to secondary postoperative tearing. More critically, with the intensifying global trend of population ageing and the widespread prevalence of metabolic syndrome, the repair of TBI injuries is facing unprecedented clinical challenges. Under these chronic degenerative pathological conditions, a cascade of failures occurs in the immune and metabolic microenvironment at the interface, manifesting as intense oxidative stress, collapse of the microvascular network, and persistent aseptic inflammation. Consequently, traditional treatment strategies involving physical suturing or the application of inert tissue engineering scaffolds have repeatedly failed in clinical translation (Wang P. et al., 2025; Gao et al., 2025).

**FIGURE 1:**
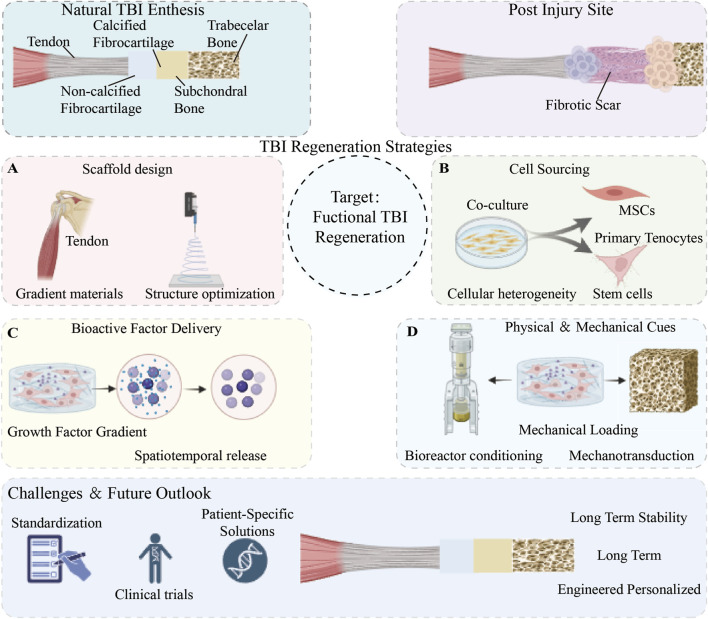
A comprehensive roadmap for engineered tendon-to-bone insertion (TBI) regeneration. The schematic contrasts the native enthesis gradient with pathological fibrotic healing, highlighting the paradigm shift toward multidimensional microenvironment reprogramming. The core bioengineering modules, encompassing intelligent scaffold design **(A)**, multi-source cell integration **(B)**, spatiotemporal factor delivery **(C)**, and biophysical cues **(D)**, synergize to restore the functional interface. The translative pathway culminates in advanced pre-clinical validation and AI-guided clinical applications.

**FIGURE 2:**
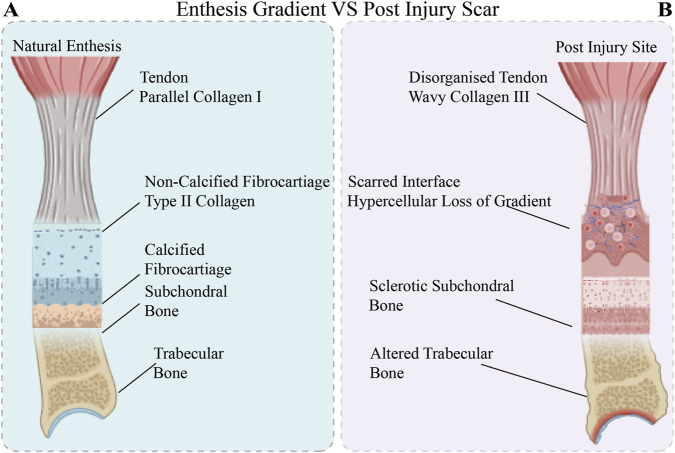
Morphological and structural divergence between the native tendon-bone interface (TBI) enthesis and pathological post-injury scar tissue. **(A)** Natural Enthesis: The healthy interface exhibits a highly organized, multi-zonal transitional gradient encompassing the tendon, non-calcified fibrocartilage, calcified fibrocartilage, and the underlying subchondral and trabecular bone. **(B)** Post-Injury Scar: Healing under pathological conditions leads to a catastrophic collapse of the native transitional structure. This scarred interface is hallmarked by a disorganized tendon featuring chaotic, wavy Type III collagen deposition, a pronounced hypercellular microenvironment with a complete loss of the compositional gradient, and underlying degenerative changes including sclerotic subchondral bone and altered trabecular bone architecture.

In recent years, the field of tissue regeneration has undergone a profound paradigm shift (Figure 1): the root cause of structural and functional integration failure in TBI is not merely insufficient mechanical fixation, but rather the loss of the local immune microenvironment’s ability to spatially and temporally orchestrate stem cell fate and matrix remodelling. In the context of ageing or chronic disease, the massive accumulation of senescent cells within the microenvironment releases the senescence-associated secretory phenotype (SASP), thereby constructing a local “inflammatory senescence” network (Wang P. et al., 2025; Healey, 2024). This pathological baseline not only blocks the pathway for macrophages to polarise towards the pro-repair M2 phenotype, but also traps resident tendon stem cells in epigenetic silencing and metabolic exhaustion (Gao et al., 2025). Furthermore, uncontrolled mechanical transduction and degenerated peripheral sensory innervation further sever the “mechanical-electrical” and “neuro-immune” communication bridges essential for maintaining homeostasis in interfacial tissues (Wang L. et al., 2025; Lu et al., 2024). Consequently, determining how to reverse this extremely adverse immunometabolic microenvironment at a fundamental level has become the pivotal challenge in overcoming the difficulties in clinical translation of traditional inert physical scaffolds, which stem from the healing challenges associated with chronic degenerative TBI. To gain a deeper understanding of the histological and biomechanical foundations of TBI healing failure, we must first clarify the fundamental differences between ideal natural attachment sites and pathological healing. As shown in Figure 2, natural TBI possesses an extremely refined structure, composition, and mechanical gradient, whereas injury often leads to the loss of this gradient and the proliferation of disorganised scar tissue. This morphological collapse is, in essence, a macroscopic manifestation of the cascade failure of the local microenvironment and cellular regulatory networks, prompting us to seek breakthroughs in deeper-level immune and metabolic mechanisms.

Against this backdrop, “bone immunology” has emerged as a highly forward-looking interdisciplinary field. This discipline has completely abandoned the traditional concept of viewing biomaterials merely as passive structural supports, instead focusing on the development of a new generation of smart biomaterials and micro-nano delivery platforms capable of actively reprogramming the host’s immune and metabolic networks (Liu W. et al., 2026). Through the precise integration of piezoelectric transducer nanofibres, asymmetric microfluidic dual-ion matrices, targeted senolytics, and engineered stem cell exosomes, these cutting-edge intervention strategies not only enable the efficient spatiotemporal penetration of therapeutic molecules within a dense ischaemic matrix, but also achieve comprehensive control over the multidimensional regenerative cascade of TBI, ranging from the subcellular level of mitochondrial energy metabolism rescue and single-cell level macrophage phenotype reversal, to the macroscopic anatomical level of vascular and neural re-innervation.

However, translating these highly complex immunological engineering strategies into widely applicable clinical therapies necessitates validation across species and biological systems. Traditional two-dimensional static cell cultures and conventional rodent models have inherent technical limitations in simulating complex human mechanical loading patterns, the progression of chronic diseases, and three-dimensional spatial heterogeneity. To build a robust translational bridge, modern preclinical evaluation systems are moving comprehensively towards advanced biomimetic microphysiological systems. Three-dimensional anchorage-dependent organoids, microfluidic organ-on-a-chip devices incorporating circulatory fluid dynamics, and spatial transcriptomics capable of high-resolution decoding of *in situ* molecular maps are collectively constructing a high-fidelity, multidimensional preclinical validation matrix (Liu et al., 2025a; Leung et al., 2022).

This review aims to systematically summarise the latest advances and future trajectories of TBI regeneration in the context of chronic degenerative diseases. We will first delve into the mechanisms of immune microenvironment collapse triggered by ageing and metabolic disorders from the perspective of bone immunology; subsequently, we will explore in detail novel smart biomaterials and delivery engineering designed to reverse macrophage polarisation and reshape mechanotransduction and the neuro-immune axis; Next, we will focus on the pivotal enabling role of advanced preclinical models in cross-scale validation and the acceleration of clinical translation. Finally, this paper offers a forward-looking perspective on the refinement of closed-loop dynamic intelligent response systems and the prospects for AI-assisted personalised regenerative medicine, with the aim of providing comprehensive and profound theoretical guidance and a technical blueprint for the precise repair of the next-generation tendon-bone interface.

## 2 Characteristics of the pathological immune microenvironment in the context of chronic diseases

### 2.1 Ageing: A multidimensional network of inflammatory ageing, cellular senescence, and mitochondrial dysfunction

In the fields of orthopaedic sports medicine and tissue engineering, ageing has been established as the most significant independent risk factor for the failure of biological integration at the tendon-bone interface (TBI) (Peng et al., 2026; Shi et al., 2025). In contrast to the highly ordered, self-limiting repair cascade observed in young organisms following acute trauma, the TBI microenvironment in ageing organisms is subject to profound synergistic disruption caused by inflammaging, cellular senescence, and mitochondrial metabolic dysfunction (Peng et al., 2026; Bahat et al., 2025). At the systemic level, inflammaging manifests as a low-grade, sterile, and persistent state of chronic immune activation (Peng et al., 2026). This pathological baseline fundamentally reshapes the immune landscape at the injury site, particularly by profoundly disrupting the local polarisation dynamics of macrophages (Bahat et al., 2025; Noga et al., 2024). During the healing process in healthy individuals, macrophages rapidly transition from the pro-inflammatory M1 phenotype to the pro-repair M2 phenotype following early debridement; however, in the ageing microenvironment, persistent inflammatory stimuli “lock” macrophages at the epigenetic level through the excessive activation of classical inflammatory signalling pathways such as nuclear factor-κB (NF-κB) and mitogen-activated protein kinase (MAPK), causing them to remain in the M1 phenotype for prolonged periods (Chen et al., 2026). This abnormal macrophage retention not only leads to the sustained release of tumour necrosis factor-α (TNF-α) and interleukin-1β (IL-1β), but also directly inhibits the self-renewal and osteogenic/tendinogenic differentiation potential of tendon progenitor cells (TDSCs) and bone marrow mesenchymal stem cells (BMSCs) via paracrine pathways, thereby completely blocking the physiological transition of the interface tissue from the proliferation to the remodelling phase (TDSCs) and bone marrow mesenchymal stem cells (BMSCs), thereby completely blocking the physiological transition of the interface tissue into the proliferation and remodelling phases (Qian et al., 2025).

At the cellular level, the pathological accumulation of p16INK4a^+^ and p21CIP1^+^ senescent cells at the tendon-bone interface, which accompanies local tissue ageing, constitutes the key biological engine driving the irreversible degradation of the extracellular matrix (ECM) (Noga et al., 2024; Ajoolabady et al., 2025). These cells, which are in a state of permanent cell cycle arrest, are not merely quiescent and harmless; rather, they actively disrupt the surrounding healthy tissue network by releasing a highly heterogeneous senescence-associated secretory phenotype (SASP) (Wang Z. et al., 2025). The SASP network is not only rich in pro-inflammatory cytokines but also contains a large number of abnormally overexpressed matrix metalloproteinases, particularly MMP-1 and MMP-13. Recent studies indicate that SASP factors abnormally disrupt the PI3K/AKT signalling pathway in surrounding healthy cells, triggering uncontrolled cascading degradation reactions. This leads to the rapid collapse of newly formed and immature Type I and Type III collagen scaffolds, resulting in isotropic disorganisation of collagen fibre alignment (Desdín-Micó et al., 2020). This molecular-level ECM disruption directly deprives the interstitium of the physical and morphological prerequisites for forming a dense “mineralised fibrocartilage band.” In a rotator cuff tear reconstruction model in aged rats, it can be clearly observed that, due to the deterioration of the tissue microenvironment mediated by SASP, tendon-bone healing often stagnates at the stage of fibrovascular scar tissue with extremely poor mechanical properties, making it difficult to achieve complete regeneration of the natural gradient structure and biomechanical function (Cai et al., 2023). Addressing this critical mechanism, a biomimetic nanofibre system derived from natural fish swim bladders has been developed in recent years. Owing to its exceptional ability to modulate ECM-responsive signalling, it offers a highly promising physical intervention strategy for counteracting age-induced matrix degradation.

More critically, cellular senescence and subcellular mitochondrial dysfunction form a mutually reinforcing, vicious positive feedback loop within the pathological microenvironment of TBI (Zhang Y. et al., 2025). In aged BMSCs and TDSCs, the structural integrity of the mitochondrial electron transport chain is compromised, and mitophagy function is severely impaired, leading to massive electron leakage and the generation of superoxide anions, which in turn cause a pathological surge in reactive oxygen species (ROS) levels within the local microenvironment (Chen et al., 2026; Zhang Y. et al., 2025). Excessive free ROS not only causes direct DNA and lipid peroxidation damage to local healthy cells, accelerating the depletion of the stem cell pool, but also acts as a potent intracellular second messenger, further amplifying and consolidating pro-inflammatory signals within the MAPK/NF-κB pathway in macrophages (Bahat et al., 2025; Zhang Y. et al., 2025). In age-related degenerative conditions complicated by osteoporosis, this extreme state of oxidative stress severely disrupts the dynamic balance between osteoblast and osteoclast metabolism, accelerating the erosion of subchondral bone microstructure (Ding et al., 2024). To break this deadlock, cutting-edge biomaterial design is shifting from passive support towards active genomic and metabolomic interventions. For example, researchers have developed a liposomal delivery system loaded with bioactive siRNA; by precisely silencing specific pathological targets in osteoporosis models, they have successfully restored the tendon-bone healing potential in aged mice (Qian et al., 2025). In summary, modern bone immunological engineering strategies must transcend the limitations of traditional mechanical fixation alone. By integrating nanomaterials, ion regulation, and gene delivery platforms to precisely modulate macrophage polarisation, target the clearance of senescent cells, and reshape mitochondrial metabolic homeostasis, it is possible to fundamentally reverse the ageing TBI microenvironment and open up entirely new translational medical pathways for functional tissue reconstruction in elderly patients.

### 2.2 Diabetes: The AGE-RAGE axis and metabolic paralysis

Among the clinical challenges of orthopaedic biological reconstruction, the systemic and local metabolic disorders induced by diabetes present an extremely formidable pathological barrier to regeneration at the tendon-bone interface (TBI) (Li S. et al., 2025; Kantharia et al., 2025; Lv et al., 2026). A central feature of this chronic pathological microenvironment is the non-enzymatic glycation driven by long-term hyperglycaemia, leading to the irreversible and massive deposition of Advanced Glycation End products (AGEs) within the tendon and bone extracellular matrix (ECM) (Wu et al., 2025). The excessive accumulation of AGEs first induces abnormal intermolecular cross-linking of type I and type III collagen fibres at the physical level. This not only greatly increases the mechanical stiffness of the local matrix and significantly weakens the ultimate tensile strength of the tendon tissue, but also fundamentally eliminates the physiological topological cues required for stem cell adhesion and macrophage mechanotransduction (Li S. et al., 2025; Kanai et al., 2025). More importantly, the glycated ECM evolves into a pathological biochemical reservoir; moving beyond mere structural damage, it exerts a devastating remodelling of the local immune landscape at the site of TBI through high-affinity binding to the transmembrane receptor RAGE (Receptor for Advanced Glycation End-products) (Wu et al., 2025; Xu D. et al., 2025). RAGE is widely expressed on the surface of local macrophages and osteoprogenitor cells; its sustained cross-linking with AGEs triggers downstream, extremely intense and persistent intracellular signalling cascades, particularly the abnormal overactivation of the NF-κB and MAPK pathways (Xu D. et al., 2025; Haythorne et al., 2019). This persistent biochemical stimulation locks macrophages, which should otherwise dynamically transform during the repair process, into the pro-inflammatory M1 phenotype, resulting in a delayed resolution of inflammation. Accompanying the prolonged residence of M1 macrophages, a large number of catabolic cytokines accumulate at the interface, forming a highly cytotoxic, hard-to-heal area. In response to this pathological network, modern bone immunology has broken through the limitations of traditional physical barriers, shifting the intervention target to the source of signal transduction. By introducing nanostructures with specific receptor-interference capabilities into this microenvironment, AGE-RAGE signal transduction can be directly blocked through steric hindrance and competitive binding effects. This effectively shuts off the NF-κB inflammatory amplification switch at its source, reversing the local suppression of microvascularisation and clearing biochemical obstacles to stem cell homing (Wu et al., 2025; Zheng et al., 2025).

The prolonged overactivation of the AGE-RAGE axis further induces a profound state of “immunometabolic paralysis” within the damaged interface, thereby depriving the interface of its self-healing potential. Mechanistically, this paralysis is driven by a synergistic mechano-biochemical blockade. Physically, the abnormal matrix stiffness induced by AGE crosslinking acts as a mechanical signal that continuously upregulates glycolytic enzymes in macrophages through mechanotransduction pathways. Biochemically, persistent AGE-RAGE signaling triggers an intracellular vicious cycle, where downstream overactivation of NF-κB stabilizes HIF-1α, locking macrophages in a hyperactive aerobic glycolysis state. In addition, this highly active signaling axis triggers a burst of intracellular reactive oxygen species (ROS), directly causing structural damage to the mitochondrial electron transport chain (ETC) (Kantharia et al., 2025; Haythorne et al., 2019). Therefore, the physiological transition of macrophages from the M1 to the pro-regenerative M2 phenotype completely depends on the reprogramming of their energy metabolism towards mitochondrial oxidative phosphorylation (OXPHOS), and this process is completely blocked by such severe oxidative stress and mitochondrial dysfunction (Yang J. et al., 2025). To overcome this multifaceted metabolic barrier, the precise remodelling of the immune microenvironment has become a top priority. Upon introduction into the TBI reconstruction system, a dual-regulating Trojan horse nanotherapy targeting mitochondrial metabolism is able to penetrate cellular barriers to reach the mitochondria directly, dynamically forcing the energy substrate utilisation of macrophages back onto the oxidative phosphorylation pathway. This thoroughly releases the stubborn lock-in of the M1 phenotype at the molecular metabolic level and drives potent M2 polarisation (Yang J. et al., 2025). Furthermore, to maintain long-term redox homeostasis at the dynamically stressed tendon-bone interface, tissue-conformable organic selenium hydrogels with microphase-controlled release properties have demonstrated exceptional capacity for reversing the pathological microenvironment. This intelligent biomaterial network precisely conforms to the complex topological structure of the defect site, whilst, through the catalytic properties of selenium atoms, continuously neutralising and scavenging harmful free radicals erupting within the microenvironment. In doing so, it constructs an immune sanctuary free from oxidative stress, ultimately successfully reactivating the osteogenesis-vascularisation coupling and the regeneration programme of the mineralised fibrocartilage zone amidst the extreme metabolic adversity of diabetes (Zhao J. et al., 2026).

### 2.3 Rheumatoid arthritis (RA): Cytokine storm and osteoclast hyperactivity

Unlike chronic metabolic degenerative diseases such as ageing and diabetes, rheumatoid arthritis (RA) is characterised by the destruction of the tendon-bone attachment sites (entheses), which is marked by a combination of intense immune aggression and acute flare-ups (Wang R. et al., 2026; Jiang et al., 2026). In the pathological microenvironment of RA, the breakdown of systemic immune tolerance leads to autoreactive T cells, B cells, and a large number of circulating monocytes breaching the synovial vascular barrier and undergoing a cascade-like infiltration towards the tendon-bone healing interface (Liu H. et al., 2026). This process is accompanied by a dramatic reprogramming of the local metabolome; metabolite analyses in longitudinal clinical cohorts confirm that the abnormal accumulation of specific lipid and amino acid metabolites not only directly correlates with disease severity but also provides the energy substrates for the sustained progression of local inflammation (Zhu et al., 2025). Supported by this abnormal metabolic network, the immune network dominated by Th1/Th17 cell subsets and M1-polarised macrophages generates a large amount of destructive cytokines at the damaged interface. Supraphysiological doses of tumour necrosis factor-α (TNF-α), interleukin-6 (IL-6) and interleukin-17 (IL-17) not only cause macrophages to lose their plasticity to convert to the M2 phenotype, but also strongly activate fibroblast-like synoviocytes (FLS) (Wang R. et al., 2026; Huang T. et al., 2026). Highly invasive FLS secrete large amounts of collagenase and matrix metalloproteinases, indiscriminately enzymatically degrading the dense collagen matrix network at the attachment site, thereby completely dismantling the physical and topological foundations for stem cell colonisation and fibrocartilage zone remodelling (Chen J. et al., 2025).

Within the core framework of bone immunology, RA’s disruption of the homeostasis of osteogenic remodelling at the interface is particularly devastating; this is precisely the fundamental reason why traditional inert biomaterials face extremely high rates of loosening and surgical failure in such patients. In physiological tendon-bone integration, subchondral bone remodelling is highly dependent on the spatiotemporal balance of the RANKL/RANK/OPG signalling axis (He et al., 2025). However, high concentrations of TNF-α and IL-6 in the RA microenvironment synergistically upregulate RANKL expression on the surface of osteoblast precursor cells, whilst IL-17 directly drives the recruitment and multinucleation of osteoclast precursor cells via multiple paracrine pathways (Huang T. et al., 2026; He et al., 2025). This unidirectionally amplified pathological signal disrupts the dynamic equilibrium of RANKL-OPG, transforming local osteogenesis-vascularisation coupling into aggressive osteolysis, which clinically often manifests as rapid cystic changes and irreversible bone loss at muscle-tendon insertion sites, such as the greater tubercle (Liu H. et al., 2026). Against this osteolytic backdrop of extreme turnover rates and intense oxidative stress, any conventional suture anchor or tissue-engineered scaffold lacking the capacity for active immunological intervention is inevitably rapidly phagocytosed or degraded by hyperactive osteoclasts, rendering regeneration strategies relying solely on physical reinforcement completely ineffective in the face of the pathological onslaught of RA (Jiang et al., 2026; Chen J. et al., 2025).

To fundamentally reverse this inflammatory and osteolytic microenvironment, modern bone immunology engineering is dedicated to upgrading intervention targets from passive structural replacement to fundamental biochemical reprogramming of immune and metabolic networks. Targeting the characteristic acidity and high oxidative stress of the RA microenvironment, stimulus-responsive multifunctional smart nanosystems offer a breakthrough approach for restoring local immune homeostasis. Precisely engineered two-dimensional nanosheets, upon implantation at the pathological interface, can sensitively detect pH fluctuations in the microenvironment and undergo dynamic hydrolysis; This hydrolysis cascade not only precisely neutralises potent pro-inflammatory acidic signals *in situ*, but also disrupts RANKL-mediated intracellular signalling through specific steric hindrance, thereby effectively blocking the activation pathway of osteoclast precursor cells and curbing invasive bone resorption at the interface at its source (Ji et al., 2025). Concurrently, to counteract the burst of cytotoxic reactive oxygen species (ROS) during RA flare-ups, a self-assembled array of myricetin-arginine conjugate nanozymes has been deeply integrated into the interface repair platform. Leveraging its exceptional cascade catalytic activity, this biomimetic system acts like a molecular sponge, continuously scavenging excess free radicals in the damaged area. By regulating intracellular redox homeostasis, it forcibly breaks the M1 phenotypic lock of macrophages, inducing pro-regenerative M2 phenotypic polarisation (Hong et al., 2026). These advanced material strategies, possessing targeted detoxification and immune remodelling capabilities, not only successfully halted the ongoing erosion of the attachment site matrix by the osteoclastic storm but also established a safe “immune sanctuary” for the tendon stem cell pool and newly mineralised cartilage within the highly destructive RA joint, thereby truly returning the initiative for tissue integration to the tendon-bone interface (Ji et al., 2025; Hong et al., 2026).

**FIGURE 3:**
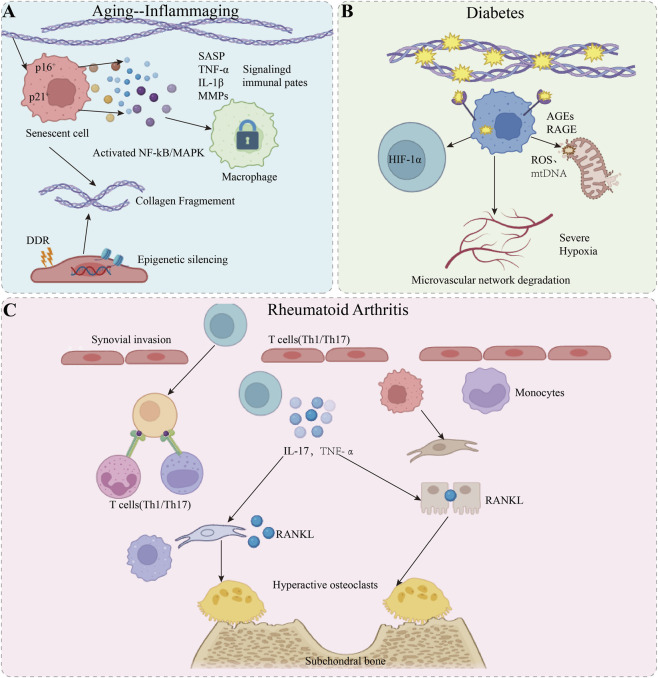
Schematic representation of the pathological immune-metabolic microenvironment at the tendon-bone interface (TBI) across distinct chronic conditions. **(A)** Ageing: The accumulation of senescent cells and the secretion of senescence-associated secretory phenotype (SASP) factors drive inflammaging and irreversibly lock macrophages in a pro-inflammatory M1 phenotype. **(B)** Diabetes: The AGE-RAGE axis triggers structural matrix cross-linking and intracellular metabolic paralysis, characterized by mitochondrial dysfunction, ROS burst, and cGAS-STING activation. **(C)** Rheumatoid Arthritis: Infiltration of autoreactive T cells (Th1/Th17) and the resulting cytokine storm disrupts the RANKL/OPG balance, leading to hyperactive osteoclastogenesis and irreversible subchondral bone erosion.

To intuitively synthesize the complex cascading events described above, Figure 3 provides a comprehensive schematic of the distinct immunometabolic hallmarks, ranging from SASP-driven inflammaging to metabolic paralysis and cytokine-induced bone erosion, that define the pathological TBI microenvironment under these chronic conditions.

## 3 Immune regulatory mechanisms at the tendon-bone interface

### 3.1 Spatio-temporal organisation of macrophage heterogeneity and polarisation dynamics

Before dissecting the specific cellular players, it is imperative to rigorously define “metabolic reprogramming,” which serves as the core biochemical engine driving these regulatory mechanisms. In the context of modern osteoimmunology, metabolic reprogramming extends far beyond a simple compensatory shift in cellular adenosine triphosphate (ATP) production. Rigorously defined, it is the profound and targeted rewiring of intracellular metabolic fluxes, such as the shifting balance between aerobic glycolysis, tricarboxylic acid (TCA) cycle anaplerosis, and mitochondrial oxidative phosphorylation. Crucially, this spatial and temporal rewiring dictates cell fate because specific metabolic intermediates transcend their traditional roles as mere energy substrates. Instead, they act as potent secondary messengers and epigenetic modifiers. By directly regulating the activity of chromatin-remodeling enzymes and stabilizing key transcription factors, this metabolic rewiring fundamentally governs the transcriptional landscape, thereby mechanically dictating macrophage polarization plasticity and stem cell lineage commitment at the epigenetic level.

In the cascade of natural healing and remodelling at the tendon-bone interface (TBI), macrophages exhibit high phenotypic heterogeneity and profound environmental plasticity; their polarisation kinetics constitute a central immune hub determining the biological integration of the interface tissue (Wang P. et al., 2025; Liu W. et al., 2026). Under traditional physical suturing or suture-anchor fixation paradigms, the majority of clinical repair failures do not stem from a lack of early mechanical strength, but rather from the local immune microenvironment’s loss of the ability to spatially and temporally orchestrate the polarisation trajectories of macrophages (Liu W. et al., 2026; Li J. et al., 2024). In the early stages of acute injury, the rapid recruitment and retention of M1 macrophages constitute an indispensable physiological defence; acting as the microenvironment’s primary scavengers, they release initial chemotactic signals by phagocytosing necrotic tissue and apoptotic cells. However, successful tissue-level cascade repair relies strictly on the timely and complete conversion of the macrophage population to the pro-regenerative M2 phenotype (Wang P. et al., 2025). Under the influence of chronic pathological environments, local kinase signalling networks, particularly the nuclear factor-κB and MAPK pathways, become hyperactivated, leading to macrophages being epigenetically and metabolically locked into the pro-inflammatory M1 phenotype (Li J. et al., 2024; Zhang S. et al., 2025). This polarisation blockage directly severs the biological bridge, enabling the transition of tissue from the inflammatory phase to the proliferative phase, resulting in the interface being persistently flooded with catabolic enzymes and cytotoxic free radicals, thereby completely dismantling the microenvironment required for stem cell engraftment and mineralised matrix deposition (Zhang S. et al., 2025; Chen et al., 2024).

In response to this immunological impasse, modern bone immunology has overturned the traditional concept of viewing biomaterials as mere physical scaffolds, instead upgrading them to microenvironmental modulators capable of actively reprogramming macrophages (Gao et al., 2025; Liu W. et al., 2026). As the biochemical engine for reconstructing the tendon-bone interface, M2 macrophages play a pivotal role through their dominant paracrine factor network, which is key to driving heterogeneous tissue regeneration. High concentrations of transforming growth factor-β (TGF-β) and vascular endothelial growth factor (VEGF), secreted specifically by M2 macrophages, act synergistically to trigger collagen synthesis in fibroblasts and the ingrowth of microvascular networks, thereby providing a physical template and nutritional support for calcium and phosphorus deposition by osteoprogenitor cells; simultaneously, the released interleukin-10 (IL-10) suppresses the excessive activation of surrounding inflammatory cells via potent paracrine pathways, thereby completely closing the local inflammatory window of opportunity (Wang P. et al., 2025; Li J. et al., 2024). To forcibly initiate this regenerative programme, cutting-edge smart bio-intervention platforms deeply integrate nanotopology with biochemical ligand delivery technologies. By constructing biomimetic interfaces with specific micro- and nano-scale features or introducing highly active antioxidant nanoenzyme systems, these advanced delivery networks, once implanted into the defect site, are able to sensitively detect and *in situ* eliminate pathological excess reactive oxygen species (ROS), whilst simultaneously releasing biochemical signals to precisely upregulate the PI3K/AKT survival pathway and block M1-type pro-inflammatory signalling cascades (Gao et al., 2025; Chen et al., 2024). This finely tuned immunological orchestration at the molecular and cellular levels not only successfully breaks the inflammatory lock-in associated with chronic pathological states but also, by inducing strong M2 polarisation, fundamentally reverses abnormal peritendinous fibrosis and the resorption of microfractures. Ultimately, it provides a suitable immunological environment for the regeneration of the mineralised fibrocartilage band within a highly complex mechanical stress environment (Gao et al., 2025; Zhang S. et al., 2025).

### 3.2 Mechanical transduction: The double-edged sword effect and spatial decoupling of the Piezo1-YAP axis in the immune-matrix mechanical network

The natural tendon-bone interface (TBI) is an extreme physical microenvironment that is constantly subjected to high stress concentrations. In traditional orthopaedic reconstruction concepts, the biomechanics of the interface are often viewed merely as a metric for assessing the strength of physical fixation; However, modern bone immunology reveals that the mechanical gradient spanning the soft-hard tissue transition zone is, in fact, the core biochemical signal driving the remodelling of the local immune and metabolic microenvironment. In this process, Piezo1, a key mechanosensitive cation channel on the cell membrane, and its downstream Yes-associated protein (YAP) signalling axis constitute the central hub for converting extracellular mechanical stimuli into genomic transcription and immune regulatory signals. Under physiological conditions, the Piezo1-YAP axis governs the lineage plasticity of interfacial progenitor cells. When local matrix stiffness increases, the opening of Piezo1 channels triggers an instantaneous, high-flux influx of calcium ions (Ca^2+^), driving a robust nuclear translocation of YAP; this cascade not only directly suppresses the expression of the tendon-specific transcription factor Scleraxis (Scx), forcibly redirecting the developmental trajectory of periosteal progenitor cells towards the osteogenic programme (Wang L. et al., 2025), but also serves as an indispensable survival signal for maintaining mitochondrial bioenergetics in the endochondral ossification zone. By regulating the β-catenin/LARS2 pathway, moderate mechanical transduction ensures efficient synthesis of adenosine triphosphate (ATP) within chondrocytes and inhibits the excessive accumulation of reactive oxygen species (ROS), thereby providing a metabolic microenvironment conducive to the mineralised healing of fractures (Zhang T. et al., 2025).

**FIGURE 4:**
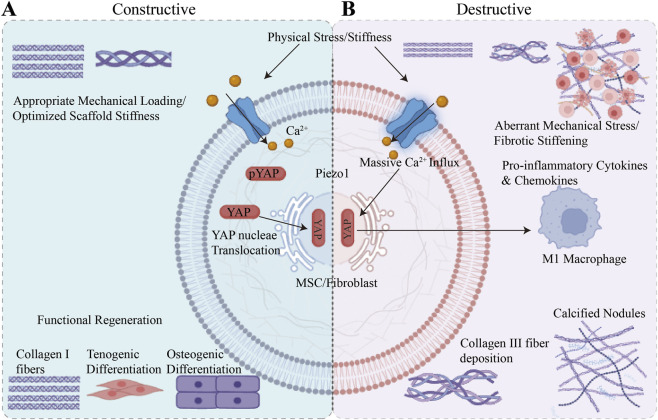
The “double-edged sword” effect of the Piezo1-YAP mechanotransduction axis in tendon-bone interface (TBI) remodeling. **(A)** Physiological and Constructive Edge: Under appropriate mechanical loading or optimized biomaterial stiffness, Piezo1 activation mediates moderate Ca^2+^ influx. This physiological signal triggers precise YAP nuclear translocation, promoting the organized deposition of Type I collagen and guiding resident stem cells toward functional tenogenic and osteogenic differentiation. **(B)** Pathological and Destructive Edge: Conversely, under aberrant mechanical stress or within a fibrotic, excessively stiff microenvironment, Piezo1 channels are chronically overactivated. The subsequent massive calcium overload and sustained YAP nuclear retention drive malignant crosstalk between fibroblasts and macrophages. This inflammatory cascade culminates in chaotic Type III collagen deposition, hypertrophic scarring, and heterotopic ossification (HO).

However, during the early stages of healing following TBI or within pathological environments characterised by abnormal stress shielding, mechanical transduction often drives the deterioration of the inflammatory microenvironment and the collapse of interfacial structures. Excessive mechanical stretching, through Piezo1-dependent calcium homeostasis imbalance, continuously induces cytoskeletal reorganisation and excessive activation of fibroblasts, directly leading to hypertrophic scarring and intractable tissue adhesions in pathological stress zones (Tu et al., 2025; He et al., 2021). A more groundbreaking immunological insight is that mechanical transduction is not limited to interactions between cells and the extracellular matrix (ECM), but is also deeply involved in physical crosstalk between immune cells and matrix cells. In a highly active pro-fibrotic microenvironment, specific macrophages can establish direct physical contact with fibroblasts via αvβ3 integrins on their surface. This microscopic intercellular contact, acting as an acute mechanical stimulus, is precisely captured by Piezo1 on the surface of fibroblasts, instantly triggering an intracellular calcium surge. Consequently, within a pathological matrix that is inherently soft and mechanically constrained, this process overcomes the limitations of biochemical ligands to initiate fibroblast-mediated matrix contraction and a pro-fibrotic network (Ezzo et al., 2024). Concurrently, sustained abnormal mechanical loading promotes deep coupling between Piezo1 and local metabolic networks; through the specific upregulation of glycolytic pathways—such as the abnormally high expression of Glut1 and aldolase—this further amplifies the infiltration of inflammatory cells, including macrophages, at the interface (Xue et al., 2025). In the degenerative TBI microenvironment associated with ageing, this uncontrolled mechanical transduction even exhibits potent cytotoxicity; its abnormal activation, via the calmodulin-dependent kinase II (CaMKII)/activator of transcription factor 3 (ATF3) signalling axis, powerfully suppressing the transcriptional activity of SLC7A11, inducing widespread ferroptosis in local matrix cells, completely blocking the reconstruction of the microvascular network and leading to severe stagnation of the healing process (Jin C. et al., 2025). To visually conceptualize this highly context-dependent mechanosensing paradigm, Figure 4 maps the “double-edged sword” effect of the Piezo1-YAP axis, contrasting its constructive physiological role in tissue regeneration with its destructive pathological cascade.

Based on a profound understanding of the double-edged sword effect exhibited by Piezo1 in physiological remodelling and pathological immuno-fibrosis, the design logic of modern bone immunomodulatory materials has achieved a paradigm shift from passive dynamic support to active mechanical signal spatial decoupling. To precisely block excessive mechanical transduction and abnormal physical contact between immune cells on the tendon parenchyma side of the interface, cutting-edge tissue engineering strategies have abandoned inert materials in favour of constructing delivery platforms with spatiotemporal responsiveness. The deep integration of a specific Piezo1 antagonist peptide (GsMTx4) into tissue-conformable hydrogel systems, such as methacrylated gelatin (GelMA), enables sustained pharmacological blockade of local abnormal Piezo1 channels following implantation at the injury site. This precision biochemical intervention, based on smart biomaterials, disrupts the mechanical transduction pathways of osteogenic and fibrogenic signalling molecules such as Apelin without compromising the macroscopic mechanical support of the tissue, thereby eliminating the malignant mechanical crosstalk between fibroblasts and macrophages at its source. Through this active immunological and mechanical reprogramming at the material interface, we have not only successfully circumvented the complication of heterotopic ossification (HO), which is highly prone to occur during TBI healing, but have also guided local progenitor cells to remodel a dense tendon matrix of extremely high purity and biomechanical tensile strength within a sanctuary protected from pathological stress interference (Tu et al., 2025; Lei et al., 2026).

### 3.3 The neuro-immune-skeletal axis: Spatiotemporal immune remodelling and bionic intervention of the sensory neurotransmitter CGRP

Within the complex tissue domain of the tendon-bone interface (TBI), simple mechanical transmission and local cellular interactions alone are insufficient to describe the full picture of tissue regeneration. One of the core breakthroughs in modern bone immunology has been the revelation of the decisive “commander-in-chief” role played by the macroscopic “brain-bone axis” and local sensory nerve endings in reshaping the immune microenvironment (Yin et al., 2025; Li R. et al., 2024). In traditional interface repair paradigms, inert mechanical fixatives often transform the implantation site into a dead space isolated from the nervous system, which explains why, in patients with chronic conditions accompanied by peripheral neuropathy—such as ageing and severe diabetes—interface tissues frequently undergo irreversible fibrotic collapse. To break this impasse, cutting-edge biomaterial intervention strategies are undergoing a fundamental shift in underlying logic, moving from passive structural filling towards the active reconstruction of the “neuro-immune-skeletal” tripartite communication network (Zhao Y. et al., 2026).

In the initial stages of physiological tissue reconstruction, the immune and nervous systems at the damaged interface exhibit highly synergistic dynamic closed-loop crosstalk. Early-recruited specific neutrophil subsets, particularly those expressing the IL-4RA+CCL2-high phenotype, are not only responsible for the initial clearance of debris but also act as neurotrophic guides, actively directing the explosive ingrowth of unmyelinated nociceptor terminals into the core of the defect by releasing potent chemokines (Qi et al., 2026). Accompanying this neural remodelling, high concentrations of neurotransmitters such as calcitonin gene-related peptide (CGRP) are released into the microenvironment, rapidly assuming control of local immune responses. By binding with high affinity to its specific receptor complex (RAMP1), CGRP triggers profound epigenetic and metabolic reversals within macrophages, forcibly interrupting pro-inflammatory signalling cascades, driving macrophage polarisation towards the pro-repair M2 phenotype, and accelerating the phagocytosis and clearance (efferocytosis) of apoptotic cells in the inflammatory zone (Lu et al., 2024). Concurrently, this neurogenic biochemical signal transcends the boundaries of immune cells to directly influence the fate of stromal cells. Through the synergistic secretion of CGRP and nerve growth factor (NGF) by sensory nerve endings, the secretory phenotype and immunosuppressive function of local fibroblasts are profoundly remodelled (Wang K. et al., 2025); furthermore, CGRP is capable of specifically activating autophagy in osteoprogenitor cells. The activation of this autophagy pathway precisely inhibits the nuclear translocation of arachidonic acid 5-lipoxygenase (5-LOX), promoting a reallocation of lipid metabolism towards the synthesis of the pro-inflammatory resolution mediator lipoxin A4 (LXA4), thereby successfully establishing a spatial coupling of immune resolution and matrix deposition in the bone remodelling zone (Qi et al., 2026).

However, in the age-related degenerative microenvironment associated with ageing, the distribution density and secretory activity of endogenous sensory neurons undergo a precipitous decline, resulting in the stem cell pool losing critical neurodifferentiation signals. In the absence of the interaction between the neurotrophin Sema3A, secreted by sensory neurons, and the neuropilin-1 (Nrp1) receptor on the surface of bone marrow mesenchymal stem cells (MSCs), the osteogenic transcriptional programme of progenitor cells is severely suppressed, leading instead to pathological fatty infiltration (Wang T. et al., 2025). To forcibly reactivate the neuro-immune axis under these extreme pathological conditions, a new generation of intelligent bio-intervention platforms has been endowed with the dual attributes of “neurotransmitter simulation and physical biomimicry.” The deep integration of biophysical transduction modules, such as pulsed electromagnetic fields (PEMFs), or electroactive nanotopological scaffolds into the healing interface enables the *in situ* reawakening of residual sensory neurons in aged tissue under non-invasive conditions, inducing the restoration of physiological pulsed release of Sema3A and CGRP (Wang T. et al., 2025). This active microenvironment orchestration strategy, which integrates neurobionics, thoroughly overcomes the limitations of traditional materials that only provide unidirectional regulation of macrophages. By reconstructing the top-level neural innervation network, it synergistically facilitates, in a top-down manner, immune attenuation, stem cell lineage commitment, and the regeneration of a pure gradient cartilage matrix, representing the cutting-edge translational direction for overcoming the challenges of tendon-bone healing under complex chronic pathologies (Zhao Y. et al., 2026; Wang T. et al., 2025). To provide a clear and structured overview of these intricate local interactions, Table 1 systematically summarizes the distinct cellular targets, physiological roles, pathological disruptions, and targeted immunoengineering strategies corresponding to the macrophage polarization, mechanotransduction, and neuro-immune-skeletal axes.

**TABLE 1:** Summary of core immune-regulatory mechanisms at the TBI and corresponding engineered intervention strategies.

Core regulatory axis	Key cellular targets	Physiological role in native healing	Pathological disruption in chronic TBI	Advanced immunoengineering interventions	References
Macrophage polarization dynamics	M1/M2 Macrophages, Fibroblasts, Osteoprogenitors	Transition to the M2 phenotype initiates TGF-β/VEGF-driven collagen synthesis, microvascular ingrowth, and robust tissue remodeling	Epigenetic “M1 locking” via NF-κB/MAPK hyperactivation; continuous ROS burst completely abrogates the transition to the proliferative phase	Antioxidant nanozymes and specific nanotopographical cues to clear ROS, activate PI3K/AKT survival pathways, and forcibly drive M2 conversion	Kantharia et al. (2025), Haythorne et al. (2019), Yang J. et al. (2025)
Piezo1-YAP mechanotransduction	Interfacial fibroblasts, Periosteal progenitor cells	Translates physical stress into YAP nuclear translocation to precisely regulate the osteogenic vs. tenogenic lineage plasticity of stem cells	Abnormal mechanical stress triggers destructive inward calcium transients, inducing malignant macrophage-fibroblast crosstalk and hypertrophic scarring	Spatiotemporal delivery of Piezo1 antagonists via smart hydrogels to decouple aberrant mechanotransduction and prevent heterotopic ossification	Zhang T. et al. (2025), Tu et al. (2025), Xue et al. (2025)
Neuro-immune-skeletal axis	Sensory nerve endings, Neutrophils, MSCs	Neuropeptides (e.g., CGRP) rapidly resolve inflammation (efferocytosis) and perfectly couple immune resolution with matrix deposition	Age/diabetes-induced sensory denervation halts Sema3A/CGRP release, severely depriving the local stem cell pool of critical neurodifferentiation signals	Electroactive scaffolds and PEMFs to reawaken residual neurons; phase-separation hydrogels for the biomimetic on-demand release of sensory neurotransmitters	Zhao Y. et al. (2026), Wang T. et al. (2025), Parker et al. (2025)

This table synthesizes the physiological roles and pathological disruptions of three core regulatory axes and their respective bioengineering intervention strategies as discussed in Section 3.

In summary, whether it be age-induced toxic paracrine effects of the SASP, diabetes-induced energy metabolism collapse, or signal transduction runaway caused by abnormal mechanical stress, these multidimensional pathological factors ultimately converge on the complete paralysis of the local immune-metabolic network. A comprehensive comparison of the specific cellular targets and macroscopic pathological outcomes of TBI regeneration across different chronic degenerative microenvironments is summarised in Table 2. These well-defined pathological mechanisms provide precise intervention coordinates for the targeted design of next-generation smart biomaterials.

**TABLE 2:** In-depth mechanistic summary of immunometabolic and mechanotransductive collapse at the TBI under chronic degenerative conditions.

Pathological trigger	Core cellular and subcellular targets	Deep molecular and epigenetic mechanisms	Immune-metabolic-mechanical cascade collapse outcomes	References
Inflammaging	Macrophages, tendon stem/progenitor cells (TSPCs), and local microvascular endothelial cells	Abnormal locking of NF-κB/MAPK pathways; DNA damage response (DDR) triggered by telomere attrition; profound epigenetic silencing of stem cells due to abnormal accumulation of repressive transcriptional marks	Toxic paracrine diffusion of senescence-associated secretory phenotype (SASP); irreversible M1 polarization locking of macrophages; comprehensive stagnation of tissue-level collagen anabolism and remodeling failure	Healey (2024), Li X. et al. (2023), Fuhrmann-Stroissnigg et al. (2017)
Diabetes/Severe Metabolic Disorders	Mitochondrial network, subchondral osteoblasts	Severe damage to the electron transport chain (ETC) and mtROS burst; imbalanced rewiring of glycolytic metabolism; non-enzymatic pathological cross-linking induced by advanced glycation end products (AGEs)	Collapse of mitochondrial bioenergetics, inducing cellular ferroptosis/apoptosis; profound microenvironmental hypoxia; significantly increased matrix brittleness, accompanied by fatal heterotopic ossification	Parker et al. (2025), Ward et al. (2017), Rondeau et al. (2024)
Abnormal Mechanical Stress and Neural Degeneration	Interfacial fibroblasts, peripheral sensory neuron endings	Overactivation of the Piezo1-YAP mechanotransduction axis induces destructive inward calcium transients; exhaustion of the synthesis and release pools for sensory neurotransmitters (e.g., CGRP, NPY)	Catastrophic conversion of cellular contractile phenotype to pro-inflammatory/pro-fibrotic synthetic phenotype; loss of immune privilege due to severed “neural-immune” communication; progressive mechanical tearing of the interfacial microstructure	Wall Shear Stress as a; Shi et al. (2026), Zhai et al. (2026)

DDR, DNA, damage response; SASP, senescence-associated secretory phenotype; mtROS, mitochondrial reactive oxygen species; ETC., electron transport chain; NPY, neuropeptide Y.

## 4 Immunoengineering strategies for the tendon-bone interface: From materials to molecules

Given the mechanisms of microenvironmental breakdown in chronic TBI and the failure of mechano-electrical-neural interactions described above, traditional physical scaffolds are no longer sufficient to meet the complex demands of regeneration. Consequently, modern bone immunology is dedicated to developing intelligent biomaterial systems capable of dynamic response and active intervention. This section will explore immunological engineering strategies for TBI in detail.

### 4.1 Nanofibre networks: The foundation for remodelling the chronic pathological microenvironment and driving advanced preclinical models

The seamless integration of the natural tendon-bone interface (TBI) relies heavily on the anisotropic topological arrangement of collagen fibre networks at the nano- and micro-scales. In chronic pathological microenvironments such as those associated with ageing, diabetes, or rheumatoid arthritis, this highly ordered matrix structure is irreversibly disrupted by matrix metalloproteinases (MMPs), causing local cells to lose the mechanotransduction and immunoregulatory cues essential for their survival. Consequently, modern bone immunology has introduced nanofibre scaffolds not only to provide mechanical support but also to reconstruct a topological sanctuary within pathological regions of the body capable of actively inducing immune remodelling. By constructing sandwich-like or radially arranged composite structures with multi-scale gradient topological features, nanofibre networks can spatially guide the heterogeneous colonisation of different immune and matrix cell populations with precision (Jin S. et al., 2025; Hao et al., 2024). In in vivo rodent TBI healing models involving diabetes or severe inflammation, simple structural biomimicry is insufficient to counteract extreme oxidative stress and vascularisation arrest. To this end, hierarchical delivery systems have been deeply integrated into the fibre networks. The hierarchical layering of polymers loaded with anti-inflammatory factors, such as glucocorticoids, with fibre networks enriched with endothelial cell derivatives enables rapid suppression of cytokine storms in the early post-implantation phase, followed by sustained release of pro-angiogenic signals during the proliferation phase (Yu et al., 2026). To address the particularly challenging issues of microcirculatory collapse and immunometabolic paralysis in diabetic models, nanoscale platforms based on metal-organic frameworks (MOFs, such as ZIF-8 and ZIF-67) have been “woven” into the interior of electrospun fibres or grown *in situ* on their surfaces in a multi-layered manner. This hierarchically structured smart scaffold can sensitively respond to local pH fluctuations within pathological tissues, enabling the micro-phase-controlled release of transition metal ions such as cobalt and zinc, alongside specific growth factors such as PDGF-BB. This forces the reactivation of macrophage M2 polarisation and the inward growth of the microvascular network under extreme metabolic stress (Wang Q. et al., 2026). When repairing TBI defects associated with severe neurodegenerative lesions, nanofibre conduits combined with endothelial cell-derived matrix coatings further demonstrate their potential to reconstruct the “neuro-immune-vascular” ternary communication network, providing a critical biochemical guideway for the directed extension of damaged peripheral nerves and the reinnervation of target organs (Yao et al., 2026).

As the scope of material interventions continues to expand, traditional static *in vitro* two-dimensional cell cultures and conventional mouse models are no longer sufficient to accurately assess the true immunomodulatory efficacy of these dynamic smart materials under complex loading conditions. This necessitates a shift in research paradigms towards advanced preclinical validation models that integrate biophysical mechanics. Within the dynamically loaded TBI microenvironment, the introduction of piezoelectric and electroactive nanofibres offers the potential to simulate the mechano-electrical coupling physiological processes found at natural interfaces. Doping highly dispersed two-dimensional graphene nanosheets into L-polylactic acid (PLLA) significantly enhances the β-phase crystallinity and *in situ* piezoelectric coefficient of the fibrous scaffold. Under physiological loads and minor deformations, such scaffolds can spontaneously generate microelectric fields, directly influencing the intracellular calcium homeostasis of periosteal progenitor cells and macrophages (Feng et al., 2025). Furthermore, flexible, electroactive nanofibres with a core-shell structure have been developed and applied to bridge damaged interfaces; these not only stably conduct nerve impulses but also activate the release of endogenous neurotransmitters through *in situ* electrical stimulation (Sun et al., 2026). These networks of electrophysiologically active nanofibres not only demonstrate exceptional regenerative properties in small animals, but their significance extends further in that they provide the ideal biomimetic matrix for the construction of next-generation microfluidic organ-on-a-chip systems. By integrating these piezoelectric or electroactive fibre membranes into the microchambers of microfluidic chips, researchers are able to precisely simulate the local fluid shear forces, mechanical traction, and electrophysiological pulses associated with TBI *in vitro*. This enables the decoding, with unprecedented resolution, of the real-time polarisation and metabolic communication between macrophages, neurons, and stem cells under various electromechanical stimuli, within a high-fidelity environment free from the complex interferences of living organisms.

To ultimately bridge the translational gap from the laboratory to the clinic, the spatial intervention precision of advanced nanofibre materials must be rigorously validated in large-animal preclinical models that closely approximate human anatomical and biomechanical characteristics. Traditional two-dimensional electrospun membranes often face limitations in conformational matching when addressing TBI defects characterised by complex three-dimensional gradients and irregular anatomical morphology. Cutting-edge manufacturing technologies that overcome this bottleneck transform highly biomimetic nanofibre microspheres into 3D-printable bio-inks, successfully constructing hierarchical porous scaffolds that combine macroscopically customised morphology with microscopically fully permeable channels (Hu et al., 2025). This three-dimensional nanofibre scaffold not only perfectly matches the physical topology of the damaged attachment site but also provides a three-dimensional developmental scaffold for the *in vitro* construction of spatially heterogeneous attachment site organoids. Furthermore, to enable active intervention in the healing timeline within living large animals, advanced near-infrared (NIR) light-responsive phase-change particles have been precisely encapsulated within the nanofibre scaffold. In a large-animal porcine trauma model that closely mimics human immune characteristics and skin/bone mechanics, this system achieved the spatiotemporal sequential release of various bioactive molecules, including epidermal growth factor (EGF) and vascular endothelial growth factor (VEGF), through non-invasive triggering by an external light source. This intelligent intervention strategy, validated in large animal models, which forcibly shuts down inflammatory cascades by dynamically activating the PI3K-Akt and MAPK survival pathways (Zhang X. et al., 2025), not only fundamentally validates the feasibility of spatiotemporal immune orchestration within complex organisms, but also signifies that nanofibre-based bone immunology has transcended the limitations of basic mechanism research and is steadily advancing towards the stage of precision clinical translation for chronic degenerative bone diseases.

### 4.2 Piezoelectric smart hydrogels: A bionic matrix driving mechanomechanical-metabolic coupling and neuroimmune reorganisation

Within the complex biomechanical environment of the natural tendon-bone interface (TBI), the extracellular matrix, rich in anisotropic collagen, not only functions as a mechanical transducer but also exhibits significant intrinsic piezoelectric effects. This physical property—the conversion of physiological mechanical stress into local microelectric fields—serves as the underlying driving force for maintaining tissue homeostasis and guiding stem cell development. However, under chronic pathological conditions such as ageing or severe metabolic disorders, the irreversible degradation of the collagen network completely destroys this natural mechano-electrical coupling hub. To forcibly reactivate this core transmission axis in experimental models and living tissues, modern bone immunology has deeply integrated piezoelectric nanomaterials—such as poly-L-lactic acid nanofibres or heterojunction-enhanced piezoelectric crystals—with flexible hydrogel networks. The ultimate goal of this smart material design is to provide an active matrix with “mechanical-electrical-biochemical” signal conversion capabilities for constructing advanced preclinical models capable of simulating complex human pathophysiology. When implanted into the affected area, these hydrogels can generate *in situ* biomimetic microelectric fields matching those of natural tissue upon undergoing minute mechanical deformations or receiving low-intensity pulsed ultrasound (LIPUS) stimulation. These dynamic electrical signals have been demonstrated to penetrate deep into cells, where they specifically activate key intracellular survival signalling pathways such as PI3K/AKT and FAK/AKT. This enables the stepwise and highly efficient scavenging of reactive oxygen species (ROS) from cells under extreme oxidative stress, thereby powerfully driving the epigenetic polarisation of macrophages towards the pro-repair M2 phenotype, achieving a deep coupling of anti-inflammatory immunity and tissue regeneration within the *in vivo* microenvironment (Liu et al., 2025b; Zhou Q. et al., 2026; Zhou D. et al., 2026; Luo J. et al., 2025; Vinikoor et al., 2023).

In extreme TBI microenvironments, such as those associated with diabetes and severe energy metabolism collapse, electrical stimulation alone is insufficient to reverse cellular senescence. Addressing this pathological challenge, cutting-edge bioelectronic design has endowed piezoelectric hydrogels with profound metabolic reprogramming capabilities. By integrating piezoelectric and mechanoredox nanomaterials into biomimetic hydrogels, these systems transcend simple signal generation. Specifically, ultrasound-induced mechanical deformation generates built-in electric fields, driving the continuous separation of electron-hole pairs, akin to piezo-catalytic reactions under dynamic stimuli. In the glucose-rich diabetic microenvironment, ambient excess glucose uniquely acts as a sacrificial hole scavenger, undergoing *in situ* piezocatalytic oxidation (Wang J. et al., 2025; Dong et al., 2026). Concurrently, the accumulated conduction band electrons reduce local water molecules to produce highly bioactive hydrogen (H_2_). This coupled mechanochemical mechanism brilliantly kills two birds with one stone: it consumes excess glucose *in situ* to correct local metabolic imbalances, while the deep-penetrating H_2_ acts as a highly selective antioxidant. The generated H_2_ neutralizes toxic reactive oxygen species (ROS) and acts as a signaling messenger to potently enhance PINK1/Parkin-mediated mitophagy pathways in damaged cells, effectively mitigating the pro-inflammatory cGAS-STING cascade. By promptly clearing dysfunctional mitochondria, this material completely breaks the vicious feedback loop of continuous oxidative stress, thereby reconstructing an efficient energy synthesis centre for progenitor cells at the interface that are deeply mired in a metabolic crisis (Liu et al., 2025b; Liu et al., 2025c; Cui et al., 2025).

**FIGURE 5:**
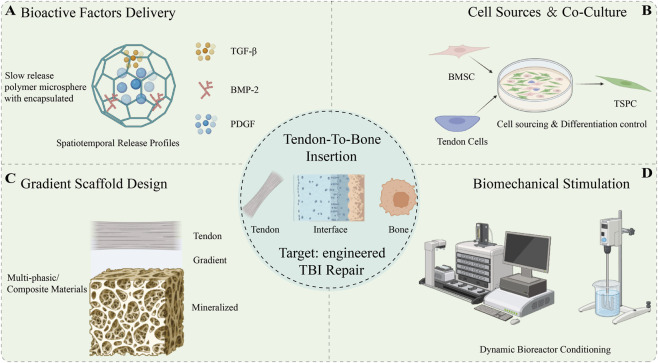
Multidimensional bioengineering strategies for TBI microenvironment reprogramming. Successful functional regeneration necessitates a synergistic approach integrating four core interventions: **(A)** multi-phasic and topologically optimized scaffold architectures; **(B)** precision delivery of bioactive factors with spatiotemporal release kinetics; **(C)** advanced cell sourcing, including stem cell co-culture systems; and **(D)** the application of dynamic physical and mechanical cues to dictate mechanotransduction. This integrated continuum aims to reverse pathological microenvironments and guide targeted tissue maturation.

Concurrently, this microscopic “mechanical-electrical-bioenergetic” multi-level conversion effect is further extended to the reconstruction of the interfacial neural network. Under ultrasonic stimulation, the high-throughput calcium influx induced by the piezoelectric hydrogel not only upregulates ATP synthase subunits and promotes mitochondrial network fusion (mediated by MFN/OPA1) (Shen et al., 2025), but also acts as an immuno-piezoelectric transducer, reversing the pro-inflammatory phenotype of microglia/macrophages in the traumatic microenvironment, thereby opening an immune-privileged pathway for the ingrowth of neural stem cells and peripheral sensory nerve fibres (Liang et al., 2026). More ingeniously, relying on the piezoelectric microfields generated by the material, specific peptides within the hydrogel can undergo liquid-liquid phase separation (LLPS) under physiological loads. These phase-separated aggregates, precisely triggered by electrical stimulation, enable the on-demand and sustained release of key sensory neurotransmitters such as neuropeptide Y (NPY), thereby perfectly reconstructing the “neuro-immune-skeletal” communication network that determines the quality of TBI healing at both the physical and biochemical levels (Zhai et al., 2026). Given the complex pathological logic and the “neuro-immune-skeletal” communication mechanism described above, single inert materials can no longer meet regenerative requirements. Consequently, there is an urgent need to develop comprehensive approaches capable of actively intervening in and reprogramming the extreme microenvironment. As shown in Figure 5, modern tissue engineering is undergoing a paradigm shift towards multidimensional integrated strategies. This strategy aims to fundamentally reverse the adverse microenvironment by synergistically integrating intelligent gradient scaffold design, multi-source cellular components, spatiotemporal controlled release of bioactive factors, and dynamic biomechanical stimulation, thereby driving both functional and structural regeneration of TBI.

To seamlessly translate the aforementioned exceptional molecular biological effects into macroscopic tissue reconstruction at the anatomical level, the physical topology and mechanical compliance of piezoelectric hydrogels have undergone revolutionary upgrades. A major physical challenge in TBI repair is the piezoelectric failure of materials under dynamic stretching. Through an innovative “PHASE” (pre-stretching-annealing-solvent replacement) strategy, researchers have successfully developed a flexible double-network polylactic acid hydrogel with a highly oriented structure. This strategy thermodynamically and kinetically locks the polar conformation of polymer chains, allowing the material to maintain an excellent piezoelectric response even under extreme interfacial strain (Wei et al., 2026). Furthermore, to overcome the limitation of traditional piezoelectric materials that only generate pulsatile electrical signals during the instant of dynamic deformation, conductive eutectic gels based on the “piezoionicity” mechanism have been introduced. Utilizing mechanical pressure-induced asymmetric migration of anions and cations, this material can convert weak, continuous mechanical strain into an uninterrupted ionic current potential, providing an exceptionally stable electrochemical microenvironment for immune cells (Li F. et al., 2026; Xu C. et al., 2025). At the clinical translation level, to resolve the repair paradox where exogenous scar adhesion suppresses endogenous regeneration, injectable piezoelectric anti-adhesion hydrogels have demonstrated tremendous clinical application potential in large animal and rodent models. Their dense networks not only form a physical barrier to effectively block fibrous infiltration from surrounding tissues, but their internal piezoelectric microcurrents also specifically stimulate the proliferation and bidirectional differentiation of encapsulated tendon stem cells, achieving a perfect balance between anti-adhesion and endogenous repair within narrow and complex anatomical spaces (Luo R. et al., 2025). Ultimately, relying on high-precision 3D printing technology, the new generation of piezoelectric scaffolds has achieved comprehensive customization from microscopic biomimetic topology to macroscopic multi-dimensional electromechanical gradients (Huang X. et al., 2026). As elegantly highlighted in recent comprehensive reviews, the structural and functional heterogeneity of the osteochondral and tendon-bone interface imposes distinct, region-specific requirements for regeneration (Yang et al., 2026). 3D printing solutions perfectly address this clinical hurdle by leveraging precise structural control and tailored material deposition to orchestrate the appropriate mechanical and biochemical microenvironments across these complex transitions (Yang et al., 2026). These smart hydrogels, possessing continuous physiological gradients and dynamic adaptive capabilities, not only thoroughly break the limitations of traditional static implants but also provide the most ideal electromechanical coupling physiological basis for the assembly of next-generation advanced *in vitro* preclinical models, microfluidic organ-on-a-chip, and enthesis organoids, paving a solid bridge for accelerating the clinical translation of precision therapies targeting chronic osteoimmune metabolic degenerative diseases.

### 4.3 Smart asymmetric interfaces and targeted delivery: Advanced microfluidic underlying architectures driving organoids and in vivo models

The essence of the native tendon-bone interface (TBI) is a highly specialized, asymmetric biomechanical and biochemical transition zone. This precise gradient physical structure is utterly destroyed in chronic pathological microenvironments induced by rheumatoid arthritis or diabetes. To construct high-fidelity enthesis organoids *in vitro* or to remodel this complex structure *in situ* within advanced *in vivo* preclinical models, modern osteoimmunoengineering has completely abandoned traditional homogeneous scaffolds in favor of biomimetic Janus membranes and asymmetric hydrogel systems. The Janus interface, constructed through a multi-scale topological combinatorial assembly strategy, not only perfectly accommodates the heterogeneous mechanical demands of the TBI macroscopically but also exhibits outstanding capabilities for spatial compartmentalized intervention. Relying on specific biomimetic micro/nano-topography and potent tissue wet-adhesion properties, one side of this system can form a seamless physical anchor with the irregular subchondral bone defect substrate, driving the *in situ* reconstruction of the osteoimmune network. Meanwhile, its dorsal side exhibits extremely low interfacial adhesion, physically blocking the invasion of exogenous fibroblasts and successfully circumventing the risk of postoperative soft tissue adhesion (Li Y.-Y. et al., 2026; Li H. et al., 2025). Above this asymmetric macroscopic matrix, to precisely simulate the spatial sequestration of different metal ions by native matrices in microfluidic organ-on-a-chip systems, such as osteogenic calcium/phosphorus or tenogenic zinc/magnesium ions, cutting-edge bioelectronics have introduced programmable ionic rectification and photo-ionic synaptic-level regulatory mechanisms. Relying on unipolar hydrogel ionic diodes with electric double layer (EDL) dynamic modulation effects and biopolymer networks acting as dynamic cation reservoirs, these smart interface materials can convert external, minute electrophysiological pulses or photophysical stimuli into unidirectional ionic flows, achieving giant programmable ionic rectification of up to several orders of magnitude within artificial matrices (Jeong et al., 2025; Wang X. et al., 2026). The establishment of such dual-ion or even multi-ion microfluidic rectification systems has thoroughly overcome the problem of paracrine signal disruption caused by the free diffusion of ions in traditional *in vitro* co-culture models and *in situ* implants. This enables researchers to reproduce biochemical gradients with high fidelity *in vitro* on chips or locally *in vivo*, providing a highly translatable biochemical foundation for decoding the spatial polarization heterogeneity of macrophages and stem cell lineage differentiation (Li Y.-Y. et al., 2026; Wang X. et al., 2026).

Besides reconstructing asymmetric interfaces at macroscopic and microscopic levels, the chronic degenerative TBI microenvironment, due to the pathological fibrotic proliferation of dense collagen networks and the severe collapse of microvascular beds, constructs insurmountable physical and physiological dual barriers, resulting in extremely low deep tissue penetration rates for conventional free-form immunomodulatory factors. This delivery bottleneck is particularly prominent in large animal preclinical models with complex gross anatomical structures and depth, becoming a core constraint hindering the transition of basic osteoimmunology discoveries to clinical applications. To achieve precise immune reprogramming in deep defect areas, microfluidic technology is not only widely used in the construction of chip models but also innovatively applied to the large-scale synthesis of micro/nanorobots performing deep targeted delivery. Permanent magnetic droplet-derived microrobots (PMDMs), efficiently assembled relying on cascade tubular microfluidic technology, have demonstrated exceptional autonomous multimodal locomotion and physical penetration capabilities within highly restricted biomimetic channels and dense living soft tissues. Through the precise reconstruction of external magnetic fields, these micro/nanocarriers can seamlessly dive high payloads of immunomodulatory drugs or stem cell clusters into the core lesion area of the enthesis without destroying the residual matrix network of the TBI (Cao et al., 2025). Even more ingeniously, in the intricate and severe fibrotic microenvironments of diabetes or rheumatoid arthritis, “dual-engine” biological micro/nanorobots with autonomous homing and environmental adaptive capabilities have achieved a paradigm-shifting advantage in precise delivery. After breaching the superficial mucus and dense connective tissue barriers at the interface, such micro/nano systems can acutely sense the locally elevated concentration gradient of inflammatory chemokines and trigger an “enzyme-macrophage switch (EMS)” mechanism *in situ*. By actively “hijacking” and hitchhiking on locally infiltrating macrophages, the biomimetic robots transform themselves into intracellular encapsulated payloads, utilizing the natural inflammatory homing properties of immune cells to accomplish a “Trojan horse” style infiltration into the highly toxic deep regions of the TBI (Zhang B. et al., 2023). This ultimate intervention strategy, which deeply integrates microfluidic structural engineering with active biomimetic nanorobots, not only completely opens up the transport channel for drugs across pathological barriers at the *in vivo* level but also successfully verifies its immense clinical translational potential to fundamentally reverse deep interfacial immunometabolic exhaustion and reboot the regeneration of dense gradient fibrocartilage in preclinical large animal trauma models that highly approximate human physiological characteristics (Cao et al., 2025; Zhang B. et al., 2023).

### 4.4 Precise orchestration and spatiotemporal delivery of biological cues

#### 4.4.1 Targeted senolytics: Cracking microenvironmental inflammatory locking via advanced preclinical models

In the chronic degenerative pathogenesis of the tendon-bone interface (TBI), cellular senescence and its derived senescence-associated secretory phenotype (SASP) constitute the core pathological cornerstone blocking tissue heterogeneous regeneration. This toxic paracrine network, composed of pro-inflammatory factors and matrix-degrading enzymes, not only completely blockades the conversion pathway of macrophages towards the M2 pro-repair phenotype but also causes nascent granulation tissue to be rapidly degraded, ultimately leading to the degeneration of the native enthesis into mechanically inferior fibrotic scar tissue (Li X. et al., 2023). Early osteoimmunoengineering attempted to reverse this predicament through the systemic application of broad-spectrum “senolytics,” such as Dasatinib combined with Quercetin (D+Q) or specific HSP90 molecular chaperone inhibitors (Healey, 2024; Fuhrmann-Stroissnigg et al., 2017). In classic rodent preclinical experimental paradigms, these small-molecule drugs indeed demonstrated the potential to attenuate local inflammatory storms and partially salvage interfacial biomechanical strength. However, the dense and severely avascular anatomical structure of the TBI severely limits the local bioavailability of systemic administration, and the non-negligible off-target toxicity poses a severe barrier to their clinical translation.

To achieve precise senescence clearance within extreme physical barriers, the design strategies of modern targeted senolytics and the evolution of preclinical evaluation models have become deeply intertwined. Cutting-edge research has begun to precisely target the unique metabolic vulnerabilities of senescent cells. For example, introducing polyunsaturated fatty acids like α-eleostearic acid can induce highly active lipid peroxidation cascades within senescent cells, achieving targeted elimination of harmful cells via the ferroptosis pathway (Zhang L. J. et al., 2026); alternatively, the combined use of SGLT2 blockers and Bcl-2/BET inhibitors forcibly disrupts the mitophagy and energy metabolism homeostasis upon which senescent cells rely for survival (Wakita et al., 2026). Even more breakthrough strategies have extracted specific lipid components from senescent cells themselves to construct biomimetic nano-delivery platforms, achieving “homologous recognition” and single-cell level high-fidelity drug delivery to senescent lesions (Ji et al., 2026). This technological leap from broad-spectrum bombardment to targeted strikes makes it difficult for traditional *in vivo* animal models to accurately track and evaluate the dynamic biochemical responses of the local microenvironment. Therefore, advanced preclinical validation platforms with high spatiotemporal resolution, particularly microfluidic organ-on-a-chip and 3D enthesis organoids, have become indispensable testing foundations. In these *in vitro* biomimetic systems, detached from the interference of systemic blood flow, researchers can monitor with unprecedented biochemical precision how targeted nano-senolytics penetrate and diffuse within dense collagen gradients, how they precisely induce apoptosis in local senescent cells, and how they simultaneously release adjacent healthy tendon stem cell pools and macrophages from inflammatory locking in real-time.

Facing the clinical pain point that the TBI is highly susceptible to secondary microenvironmental senescence under continuous mechanical stress, living cell therapies based on chimeric antigen receptor T cells (CAR-T) have been forward-lookingly introduced, aiming to construct a microenvironmental barrier with long-acting immune surveillance functions. Engineered CAR-T cells targeting the senescence marker urokinase-type plasminogen activator receptor (uPAR) can persistently reside as highly sensitive “microenvironmental patrollers” after a single local or systemic intervention. They not only potently eradicate the existing toxic SASP network but also reverse deep tissue metabolic exhaustion (Amor et al., 2020). The rigorous evaluation of the targeted safety, homing efficiency, and long-term tissue integration of these “living drugs” with self-amplification and immune memory capabilities thoroughly exceeds the carrying capacity of conventional *in vitro* cell experiments, heavily relying on complex microfluidic and *in vivo* preclinical models integrating human stem cell lineages, primary immune cell banks, and large animal anatomical features. By deeply nesting advanced metabolic targeted drugs and immune cell therapies into advanced organ-on-a-chip and large animal validation matrices, osteoimmunoengineering has not only successfully cracked the pathological deadlock of chronic degenerative tendon-bone healing but has also established a closed-loop validation paradigm from molecular mechanism resolution straight to individualized translational medicine.

#### 4.4.2 Deep integration of extracellular vesicles and smart nano-matrices: Epigenetic reprogramming validated by advanced preclinical models

After successfully stripping toxic senescent cells from the TBI microenvironment using targeted senolytics, awakening the deeply exhausted local stem cell pool and resetting the compromised immune homeostasis constitutes the critical rate-limiting step in achieving tissue heterogeneous regeneration. In TBI pathological models accompanied by advanced age, severe metabolic disorders, or chronic autoimmune diseases, local macrophage and progenitor cell populations universally exhibit profound immunosenescence, the underlying logic of which lies in genomic instability, telomere attrition, and the comprehensive collapse of mitochondrial metabolism (Li and Bai, 2025; Wang X. et al., 2025). Faced with this complex systemic degeneration, single soluble growth factors are highly prone to inactivation in the dense and ischemic enthesis matrix, prompting modern osteoimmunoengineering to comprehensively shift intervention carriers towards mesenchymal stem cell-derived small extracellular vesicles (sEVs/exosomes). As natural nanoscale biochemical reactors, exosomes secreted by juvenile tissues are enriched with specific microRNAs (miRNAs) and active proteins capable of directly combating oxidative stress and DNA damage. After being endocytosed by senescent target cells, these multi-molecular payloads can potently upregulate key anti-aging hubs like SIRT1 and tissue homeostasis maintenance pathways like TIMP1/Notch1. This not only curbs the eruption of intracellular reactive oxygen species (ROS) from the source but also profoundly reverses the epigenetics of target tissues, driving the transcriptional profile of senescent stromal cells to return to a highly active synthetic phenotype (Zhang H. et al., 2024; Zhang X. et al., 2023; Sanz-Ros et al., 2022; Yang H. et al., 2025).

To maximize the therapeutic efficacy of these nanovesicles within the inflammatory and hypoxic barriers of the TBI, bioengineering preconditioning and the development of specific cell sources have become cutting-edge breakthrough methods. In exploring specialized cell sources with extremely strong osteogenic and chondrogenic regenerative attributes, exosomes derived from antler blastema progenitor cells (ABPCs) have been pioneeringly introduced into the intervention system. These specialized stem cell vesicles not only significantly reverse the senescent phenotype of stem cells *in vitro* but also harbor extremely unique pro-mineralization bioinformation codes (Hao et al., 2025). Meanwhile, targeting severe immune microenvironments induced by conditions like rheumatoid arthritis, parental stem cells are purposefully exposed to stress conditions of 3D biomimetic mechanics, physical hypoxia, or pro-inflammatory cytokines such as interferon-γ (IFN-γ) prior to vesicle collection. This immune licensing process forces profound reconfigurations of post-transcriptional modifications in exosomes, enabling them to internally load high densities of key molecular chaperones like heat shock protein 70 (Hsp70) and specific anti-inflammatory miRNAs. Upon entering the pathological region, these engineered, refined super-vesicles can assist in the refolding of damaged proteins, directly antagonize the senescence cascades of chondrocytes, and forcibly suppress the M1 polarization locking of macrophages within an extremely short time window, completely severing the continuous inflammatory driving force that leads to enthesis collapse (Zhao et al., 2025; Liu et al., 2025d).

However, practically translating these outstanding molecular biological mechanisms into effective clinical treatments must cross two major chasms: the rapid *in vivo* clearance of free exosomes and the inability of traditional animal models to map complex human immunometabolic networks. Modern osteoimmunoengineering not only deeply integrates the aforementioned engineered exosomes with smart nanomatrices, such as piezoelectric hydrogels and dual-ion microfluidic Janus membranes to achieve spatiotemporally programmed sustained release over several weeks, but also comprehensively anchors the efficacy evaluation of this “smart material-exosome” combined intervention paradigm onto high-level preclinical validation models. *In vitro*, enthesis organ-on-a-chip and 3D organoid systems based on microfluidic technology provide a high-fidelity platform, devoid of systemic blood flow interference, for resolving the diffusion dynamics and intercellular penetration capabilities of exosomes within multi-level gradient matrices. Researchers are able to track, with single-cell resolution, how exosomes targetedly intervene with immune cells in specific regions and induce the spatial heterogeneous differentiation of fibroblastic/osteogenic precursor cells within microchambers possessing precise biochemical gradients. At the level of *in vivo* translation, because the bone remodeling rates and immunosenescence dynamics of rodents differ vastly from those of humans, these smart implants loaded with ABPC exosomes or engineered vesicles are strictly applied in aging and trauma models of non-human primates or large pigs. In these large animal models that highly approximate human physiological characteristics, it has not only been successfully verified that exosome sustained-release systems locally greatly enhance the bone mineral density of the femur and enthesis and remodel dense gradient fibrocartilage, but it has also been confirmed that they can significantly improve the host’s overall motor-cognitive function and reverse epigenetic age by several months through systemic immunomodulation (Sanz-Ros et al., 2022; Hao et al., 2025). This validation pathway, utilizing advanced organoids and large animal models as the core touchstone, thoroughly proves that the fusion strategy based on exosomes and smart nanomatrices possesses substantive clinical translational capabilities to conquer the TBI regeneration dilemma under chronic pathological conditions.

### 4.5 Mitochondrial targeting and ROS scavenging strategies: Subcellular metabolic-immune reprogramming relied on advanced preclinical models

In chronic pathological environments such as aging, severe diabetes, and rheumatoid arthritis, the immunometabolic paralysis of the tendon-bone interface (TBI) centrally erupts at the subcellular level as profound dysfunction of the mitochondrial network. Long-term sterile inflammation and the ischemic collapse of the enthesis microvascular network subject local tissues to severe hypoxia and oxidative stress, causing the mitochondrial electron transport chain to be severely damaged and continuously leak large amounts of superoxide anions. This pathological burst of endogenous mitochondrial reactive oxygen species (mtROS) not only accelerates cellular senescence but also, as a key second messenger, directly activates and solidifies pro-inflammatory signaling cascades such as NF-κB within macrophages, plunging them into irreversible M1-type polarization locking. Faced with this deep pathological mechanism, traditional systemic interventions inevitably fail due to a strict “dual-barrier” constraint. Macroscopically, the dense, avascular collagen network of the enthesis severely limits the interstitial diffusion of free drugs; microscopically, conventional antioxidants lack the transmembrane driving force required to enter resident cells and enrich within mitochondria. To definitively break through this hierarchical delivery bottleneck, modern osteoimmunoengineering logically decouples matrix penetration from subcellular targeting by employing a “spatiotemporal relay” strategy. To overcome the fundamental anatomical constraint of the avascular barrier, the active agents are not administered systemically but are encapsulated within advanced nanocarriers and delivered *in situ* via the aforementioned smart hydrogels. For extremely dense fibrotic zones, active penetration driven by “macrophage-hitchhiking” microrobots is necessitated to physically ferry the payload deep into the tissue. Only after these optimized carriers navigate the macroscopic interstitial spaces and reach the immediate pericellular vicinity is the subcellular targeting mechanism engaged. For instance, by covalently coupling a potent antioxidant ubiquinone moiety with a lipophilic dodecyltriphenylphosphonium (dTPP) cation, these engineered payloads can specifically utilize the mitochondrial transmembrane potential (Δψm) to achieve sharp accumulation against a concentration gradient by hundreds of times at the inner mitochondrial membrane rich in destructive ROS, thereby constructing the first precise biochemical defense line at the epicenter of the oxidative stress storm (Parker et al., 2025).

After successfully crossing the subcellular delivery barrier, targeted mitochondrial intervention triggers extremely profound transcriptional and metabolic reprogramming within damaged cells, which is of decisive significance for salvaging the hypoxic microenvironment of the TBI. In tissue matrices facing chronic hypoxia or ischemia-reperfusion injury, excessive mtROS directly leads to the oxidative cleavage of mitochondrial DNA (mtDNA). The mtDNA fragments escaping into the cytoplasm act as damage-associated molecular patterns (DAMPs), further initiating widespread inflammaging networks. Targeted antioxidant molecules not only efficiently scavenge toxic free radicals from the source and protect the integrity of the mitochondrial genome but also specifically activate the endogenous master antioxidant Nrf2/ARE pathway in the nucleus by relieving oxidative stress (Hu et al., 2018). This cross-spatial antioxidant crosstalk established between organelles and the nucleus greatly enhances the biological resilience of stromal cells and immune cells against the inflammatory microenvironment. An even more breakthrough mechanism lies in the fact that specific intervention in the mitochondrial respiratory chain can forcibly alter the energy metabolism foundation of pathological cells. In highly hypoxic solid tissue barriers, mitochondria-targeted metabolic regulators have been proven to significantly reduce abnormally elevated mitochondrial oxygen consumption and induce cells to undergo an adaptive glycolytic shift. This profound metabolic reprogramming not only effectively alleviates the degree of absolute hypoxia in the local microenvironment but also provides a key molecular valve for subsequently reconstructing nascent microvascular networks and driving the metabolic transition of macrophages toward a pro-repair M2 phenotype (Rondeau et al., 2024).

Effectively translating these exceedingly precise subcellular metabolic rescue mechanisms into macroscopic tissue regeneration of the TBI completely overturns the validation capabilities of traditional single-cell cultures or lower animal models. Because mitochondrial metabolic dynamics are highly coupled with the body’s systemic endocrine, immune, and aging states, evaluating the true clinical potential of such targeted strategies must deeply rely on advanced preclinical validation platforms. In exploring progressive tissue fibrosis induced by metabolic degenerative diseases, researchers utilized live mammalian models combined with severe diabetes to verify that continuous mitochondria-targeted intervention could significantly arrest the recruitment of pathogenic macrophages within the lesion area, fundamentally reversing ectopic proliferation and cicatricial collapse of microstructures (Ward et al., 2017). Similarly, in models of systemic oxidative damage induced by long-term exogenous oxidative toxicant exposure, such as environmental stress, precise ROS scavenging at the subcellular level potently protected the mitochondrial density of developing organs and successfully blocked the initiation of irreversible fibrotic programs (Sukjamnong et al., 2018). To replicate this therapeutic effect in the unique dense gradient matrix of the TBI, cutting-edge osteoimmunoengineering is precisely encapsulating mitochondria-targeted molecules within smart hydrogels or microfluidic delivery carriers possessing spatiotemporal response characteristics, and subjecting them to rigorous testing in humanized microtissue models derived from induced pluripotent stem cells (iPSCs) and large animal trauma models (Parker et al., 2025). In these microfluidic organ-on-a-chip systems, which are free from species genetic differences and highly approximate human true physiological/pathological mechanical loading, researchers can track in real-time with single-cell resolution how smart carriers penetrate scar tissue, precisely restore local mitochondrial bioenergetic homeostasis, and forcibly reprogram the polarization trajectory of resident macrophages. This research paradigm, deeply nesting subcellular metabolic regulation with advanced biomimetic preclinical models, not only provides solid mechanistic evidence for cracking the deadlock of tendon-bone healing under chronic pathology but also constructs the most core technical foundation for driving the next-generation of precision osteoimmune nanodrugs toward clinical translation.

From the macroscopic physical reconstruction of asymmetric interfaces to the subcellular rescue of mitochondrial metabolism, modern osteoimmunoengineering has constructed a multidimensional intervention matrix. To clearly illustrate this technological evolution, Table 3 systematically reviews and compares the core driving mechanisms, spatiotemporal intervention characteristics, and translational challenges faced by current mainstream advanced biomaterials and smart delivery platforms.

**TABLE 3:** Mechanistic classification of advanced osteoimmunomodulatory biomaterials and spatial delivery platforms for deep-tissue reprogramming.

Engineered intervention chassis/Carrier	Targeted biochemical signals/Physical cues	Master molecules and deep transduction pathways	Tissue-level spatiotemporal immune reprogramming effects	References
Dynamic piezoelectric bioelectronic interfaces	*In situ* ultrasound/mechano-electrical conversion microelectric fields; Neuropeptide molecules released via liquid-liquid phase separation (LLPS)	Specifically activates PI3K/AKT and FAK/AKT pro-survival pathways; targetedly upregulates PIEZO1/2 ion channels in stromal cells; promotes MFN/OPA1-mediated mitochondrial network fusion	Step-wise ROS clearance and potent M2 macrophage reprogramming; electrical stimulation triggers sustained, on-demand release of peptide condensates, precisely reconstructing the microenvironmental “neural-immune-skeletal” communication axis	Shi et al. (2026), Zhai et al. (2026), Liang et al. (2026)
Janus asymmetric microfluidic membranes	Gradient metal ions (Ca/P vs. Mg/Zn); Electric double layer (EDL) dynamic ionic currents	Relying on piezoionics mechanisms, drives asymmetric migration of anions and cations via localized applied voltage or extremely weak mechanical forces, achieving artificial synapse-level unidirectional ionic rectification	Macroscopic asymmetric biomimetic topology completely blocks exogenous fibrotic scar adhesions; microscopic programmable ionic gradients guide spatial compartmentalized polarization of macrophages and drive gradient mineralization of the natural tendon-to-bone interface	Li Y.-Y. et al. (2026), Jeong et al. (2025), Wang X. et al. (2026)
Biological micro/Nanorobots and subcellular targeted platforms	Lipid peroxidation inducers; Dodecyltriphenylphosphonium (dTPP)-MitoQ	Targets unique vulnerabilities of senescent cells, hijacking ALOX15 lipoxygenase to trigger specific ferroptosis; penetrates the bilayer membrane to activate the Nrf2/ARE antioxidant response element master pathway in the nucleus	Relies on the “enzyme-macrophage switching (EMS)” mechanism to penetrate dense scars and target extremely deep lesions; forcibly downregulates mitochondrial oxygen consumption and induces adaptive glycolytic metabolic transitions in immune cells	Parker et al. (2025), Cao et al. (2025), Zhang B. et al. (2023), Zhang L. J. et al. (2026)
Engineered stem cell exosomes and living cell therapies	Immune licensing (IFN-γ preconditioning) enriched specific miRNAs/Hsp70; uPAR-targeted receptors	Targetedly activates SIRT1/TIMP1/Notch1 tissue homeostasis maintenance hubs; triggers ALKBH5-dependent m6A RNA demethylation modifications; CAR-T cell-mediated long-acting immune surveillance	Potently reverses the transcriptional profile of senescent cells without altering the genome; assists in folding damaged proteins, suppressing macrophage inflammatory storms within an extremely short time window	Amor et al. (2020), Zhang X. et al. (2023), Zhao et al. (2025), Qin et al. (2026)

LLPS, liquid-liquid phase separation; EDL, electric double layer; EMS, enzyme-macrophage switching; ARE, antioxidant response element.

## 5 Preclinical validation and advanced disease models: Cross-scale translation from organoids to large animals

The aforementioned advanced materials and strategies have achieved satisfactory results in two-dimensional (2D) cell cultures and rodent models. However, these traditional models often struggle to accurately reflect the complex mechanical-biological coupling relationships of the human tendon-to-bone interface and the specific responses of the human immune system. This makes translating these achievements difficult. To break through this bottleneck, the field is accelerating the construction of highly biomimetic preclinical validation models.

### 5.1 Enthesis organoids: Advanced in vitro biomimetic validation for bridging species differences and reconstructing spatiotemporal heterogeneity

In chronic metabolic diseases and aging environments, the immunometabolic network of the tendon-to-bone interface (TBI) exhibits a complex cascade of destruction. Traditional two-dimensional cell culture systems and conventional rodent models present insurmountable gaps in species genetics and physical space when simulating the unique gradient anatomical characteristics, precise multidimensional mechanical loading patterns, and intricate immune-stromal cell crosstalk of the human TBI. This limitation in model dimensions leads to a significant number of intelligent biomaterials and immunomodulatory drugs, which perform excellently in basic experiments, encountering translational failures upon entering human clinical trials. To completely break down this validation barrier between basic mechanistic research and clinical translation, modern osteoimmunoengineering is leveraging stem cell biology and advanced manufacturing technologies to drive a fundamental paradigm shift in the preclinical evaluation system toward musculoskeletal organoids (Liu et al., 2025a; Chen W. et al., 2025).

As a new generation of advanced *in vitro* preclinical models, TBI organoids have completely transcended the early stage of simple cell aggregates, evolving into three-dimensional biomimetic complexes capable of replicating the developmental trajectories and pathological evolution laws of human native tissues with high fidelity. To precisely reconstruct the spatial heterogeneity of the smooth transition from flexible tendon to dense calcified bone at the natural enthesis in in vitro models, high-precision 3D bioprinting technology has been introduced into organoid engineering as a core construction tool. Through the multi-scale, spatially programmed arrangement of extracellular matrix (ECM) inks with varying moduli and specific precursor cell populations, this technology has successfully constructed miniature joint and enthesis organoid tissues that possess specific gross morphologies, physical-mechanical gradients, and biochemical signaling cues. Such microphysiological systems with high spatial fidelity provide an irreplaceable *in vitro* biomimetic validation paradigm for high-throughput screening of immunomodulatory drugs and evaluating the targeted penetrability of personalized intelligent materials (Zhang Y. et al., 2024). Within this complex three-dimensional architecture, guiding a single-source stem cell population to undergo spatially specific multidirectional differentiation toward osteogenic, chondrogenic, and tenogenic lineages is the core technical barrier to reconstructing the gradient fibrocartilage transition zone. Cutting-edge biomechanical designs have deeply integrated biomimetic matrices with tunable moduli, such as methacrylated gelatin (GelMA), into the assembly process of organoids. By finely tuning the cross-linking network and microscopic physical topology of the matrix, this hydrogel microenvironment can exert specific spatial mechanical stresses on the encapsulated bone marrow mesenchymal stem cells (BMSCs) from the bottom up. Upon sensing this physical signal, the cell membrane precisely triggers the nuclear translocation of the YAP/TEAD4 transcriptional coactivator signaling axis, the intracellular mechanotransduction hub. This mechanotransduction cascade, driven entirely by the physical properties of the material, unlocks the lineage differentiation potential of healthy stem cells without the need for high concentrations of exogenous growth factors, successfully achieving coordinated development within an isolated, ideal organoid culture pool. However, it is crucial to delineate this physiological baseline from the chronic pathological environments discussed earlier. In severe *in vivo* scenarios like aging or diabetes, resident stem cells are trapped in profound epigenetic silencing and immunometabolic exhaustion, rendering their intrinsic mechanosensing pathways largely unresponsive. Therefore, the logical progression of TBI regeneration strictly dictates a “two-stage” rescue paradigm: the highly concentrated biochemical cues and engineered exosomes act as an indispensable prerequisite to “reset” the cellular baseline and clear the toxic inflammatory niche. Only after cellular plasticity is biochemically restored can these stem cells accurately sense and respond to the spatial mechanical stresses provided by the scaffold to undergo proper lineage stratification (Luo P. et al., 2025).

To transition from microscopic histological *in vitro* models to the *in vivo* repair of gross anatomical defects, the scaling up of organoids and dynamic physiological culturing constitute key bridges to living large animal models. Breaking through the millimeter-scale size limits of traditional spheroid organoids, the tissue engineering community has successfully developed transplantable self-assembling tendon organoids with lengths exceeding the centimeter scale. These macroscopic engineered tissues not only maintain extremely high cell viability and pure tendon-specific marker expression *in vitro* over the long term but also, relying on their exceptionally vigorous endogenous ECM secretion and highly ordered collagen arrays, achieve localized cell retention rates several times higher than conventional stem cell suspensions when bridging large segmental tendon-bone defects in animals directly as “living biopatches.” This fundamentally drives the efficient remodeling of dense regenerative tissue (Fang et al., 2025). However, because the matrix homeostasis and immune privilege of the natural TBI are highly dependent on the host’s continuous three-dimensional mechanical loading, subjecting macroscopic organoids to static incubators for prolonged periods causes them to rapidly lose their biomechanical properties and undergo spontaneous degeneration. To replicate the host’s dynamic loading microenvironment with high fidelity *in vitro*, interdisciplinary research has deeply physically coupled flexible bioreactors with highly biomimetic humanoid robot systems. By directly anchoring the large-scale organoids under cultivation onto the biomimetic shoulder/knee mechanical axes of the robot, the system can exert multidimensional physiological or pathological mechanical stretching on the extracellular matrix, including abduction, adduction, and rotation, which closely mimic true human kinematic parameters. This highly dynamic biomimetic mechanical stress rewires the transcriptomic profile of cells within the organoids and accelerates the mechanical maturation of the matrix (Mouthuy et al., 2022). Furthermore, when applied in combination with the piezoelectric biointerfaces discussed earlier, this setup can convert macroscopic three-dimensional deformations into dynamic microelectric field pulses *in situ* within the organoids. This cutting-edge “mechano-electric” coupled organ model provides the ultimate physical testing foundation for precisely simulating the immune-mechanical crosstalk between macrophages and mechanical stress *in vitro* (Shi et al., 2026).

Ultimately, these highly integrated enthesis organoids provide a preclinical disease mapping platform for decoding and reversing chronic degenerative pathologies at the epigenetic level. In pathogenic microenvironments of aging or severe metabolic disorders, tendon stem cells often fall into exhaustion due to profound epigenetic silencing. Relying on in vitro-induced “senescent” tendon organoid disease models, researchers can precisely validate new-generation epigenetic reprogramming therapies in a pure environment free from the body’s complex systemic endocrine interference. By targeting and activating ALKBH5-dependent m6A RNA demethylase activity, studies have conclusively demonstrated in organoid models that abnormally accumulated repressive transcriptional marks in senescent cells can be forcibly erased without altering the underlying genomic sequence. This spatiotemporal epigenetic reprogramming achieved within the 3D organoid system extremely efficiently extinguishes the tissue’s inflammaging phenotype (SASP), comprehensively awakens the metabolic vitality of stem cells, and achieves true functional tissue rejuvenation *in vitro* (Qin et al., 2026). Establishing this macroscopic organoid system (which encompasses 3D intelligent printing, robotic mechanobiology, and epigenetic pathological regulation) as the core testing ground for osteoimmune interventions ensures that all newly developed intelligent nanodelivery platforms and immune intervention macromolecules have undergone the most rigorous cross-scale efficacy and safety evaluations in the highest-fidelity humanized complex physiological systems before entering expensive and ethically constrained large animal or human clinical trials (Liu et al., 2025a; Chen W. et al., 2025).

### 5.2 Microfluidic organ-on-a-chip: Reconstructing dynamic mechano-electric and vascularized barriers

Although three-dimensional enthesis organoids have achieved breakthrough progress in reconstructing cellular heterogeneity and spatial stratification, the maintenance of homeostasis in the natural TBI heavily relies on continuous dynamic mechanical stress, complex interstitial fluid shear stress, and extremely precise microvascular immune exudation networks. Traditional static organoid culture systems possess inherent flaws in simulating microcirculatory damage and immune cell chemotactic cascades within chronic degenerative microenvironments (such as diabetes or rheumatoid arthritis) due to a lack of closed-loop fluid perfusion and precise physical stress loading capabilities. To bridge this gap between biology and engineering, modern osteoimmunoengineering introduces microfluidic organ-on-a-chip technology into the TBI regeneration validation sequence, constructing dynamic, high-fidelity preclinical models capable of precisely manipulating microscopic physical and biochemical gradients (Leung et al., 2022). By applying advanced polymer molding techniques at the micrometer scale, organ-on-a-chip platforms can highly restore the geometric topology of natural tissues, providing a completely new validation platform for decoding the “immune-matrix” environment under complex pathological states.

In reconstructing the spatial anatomical heterogeneity of the TBI, microfluidic chips based on multi-chamber compartmentalized designs exhibit advantages unmatched by traditional Petri dishes or single animal models. Cutting-edge interfacial microphysiological systems innovatively integrate electrospun aligned nanofiber yarns with microfluidic chambers to construct a tendon-bone/muscle-tendon co-culture array featuring a highly biomimetic “M-shaped” interlaced topological boundary.

This precise microfluidic architecture not only achieves precise spatial isolation of tenocytes from osteogenic/myogenic precursor cells but also allows for the free diffusion of paracrine signals and direct physical contact of microscopic synapses between heterogeneous cells through its unique micro-nanopore structure. This highly biomimetic interfacial boundary design successfully replicates the unique matrix alignment and cellular polarization characteristics of the tendon-to-bone transition zone *in vitro*. Importantly, while these isolated systems inherently lack the complex systemic endocrine circuits and multi-organ crosstalk present in a living organism, this deliberate reductionism provides a unique translational advantage: it effectively filters out systemic confounding variables. Consequently, it establishes an exceptionally rigorous and controllable *local* preclinical testing foundation for precisely evaluating the spatially specific differentiation trajectories of interface-resident cells under intervention by exogenous intelligent biomaterials, serving as an indispensable mechanistic stepping stone before advancing to complex *in vivo* large animal models. (Su et al., 2024).

Crucially, organ-on-a-chip platforms provide a visualized, dynamic validation platform for studying mechanotransduction and vascular immunodynamics of the TBI under chronic degenerative pathologies. The natural enthesis is constantly subjected to extreme stretching and fluid shear stress fields, and abnormal mechanical loading is often the core trigger for local sterile inflammation. Relying on chip systems integrated with flexible silicone membranes and pneumatic micropumps, researchers can apply precisely programmed cyclic mechanical stretching and fluid shear stress to multi-cellular culture chambers. This dynamic mechanical stimulation can activate mechanosensitive ion channels like PIEZO1 on the surface of stromal cells *in situ*, subsequently triggering transient intracellular calcium surges. This replicates the entire pathological transition process of fibroblasts or vascular smooth muscle cells from a resting contractile phenotype to a pro-fibrotic/pro-inflammatory synthetic phenotype in an *in vitro* model (Zhao et al., 2026). Building upon this dynamic mechanics, the incorporation of the vascularized chip concept further connects the immune network to local inflammatory lesions. By constructing biomimetic microvascular channels lined by primary endothelial cells on the periphery of the main culture chamber, researchers can authentically simulate chronic ischemic microenvironments or pathological states of high vascular permeability in autoimmune diseases on the chip. When fluid media containing circulating monocytes or neutrophils are continuously pumped into the vascular channels, researchers can track, with extremely high resolution in real-time, the margination, transendothelial migration, and deep chemotactic infiltration of immune cells toward the core TBI injury zone induced by specific chemokine gradients or abnormal mechanical stress. This thoroughly recreates the full picture of complex *in vivo* immune evolution *in vitro* (Khosravi and Radisic, 2026).

To break through the validation limitations of single technologies, the highest order of current orthopedic preclinical disease models is comprehensively evolving toward the deep integration of “organoid-on-a-chip.” This integrated paradigm perfectly stitches together the exceptional macroscopic three-dimensional self-assembly capabilities of organoids with the unmatched microenvironmental spatiotemporal control of microfluidic chips (Park et al., 2019; Zhao et al., 2024). Seeding TBI organoids at different developmental stages or carrying specific aging/metabolic mutations into chip chambers equipped with continuous perfusion systems and microelectrode sensing arrays not only completely solves the necrosis problem caused by limited oxygen and nutrient diffusion within large-scale organoids but also constructs an *in vitro* microphysiological closed-loop system containing all the elements of “vascular-immune-neural-skeletal” networks (Wang et al., 2024). In this highly integrated biomimetic universe, the cutting-edge osteoimmunoengineering intervention methods detailed earlier (such as piezoelectric nanoscaffolds, dual-ion microfluidic Janus membranes, targeted senolytics, and engineered exosomes) can undergo high-throughput efficacy validation and off-target toxicity screening in an extreme testing environment that highly approximates human physiological mechanisms, possessing both multidimensional electromechanical physical fields and active immune microcirculation. This organoid-on-a-chip validation network, which transcends simple *in vitro* two-dimensional cultures and complex *in vivo* animal systemic black boxes, not only provides a “microsurgical table” for decoding the mechanisms of chronic TBI immunometabolic dysregulation but also constructs an indispensable core translational bridge for accelerating next-generation precision orthopedic regenerative products targeting microenvironment reprogramming toward clinical personalized medicine (Leung et al., 2022).

### 5.3 Spatial transcriptomics: High-resolution molecular maps for decoding gradient microenvironments and evaluating material interventions

The successful regeneration of the natural TBI relies not only on the presence of specific progenitor and immune cell populations but also on the extremely precise spatial arrangement and dynamic paracrine communication of these heterogeneous cells within the transition zone from flexible tendon to rigid subchondral bone. With the maturation of advanced preclinical models such as the three-dimensional enthesis organoids and microfluidic organ-on-a-chip platforms mentioned earlier, traditional single-cell RNA sequencing (scRNA-seq) has exposed a fatal methodological flaw when validating these complex biomimetic systems: because it requires deep tissue dissociation, scRNA-seq completely destroys the original three-dimensional spatial coordinates of the cells. This makes it impossible for researchers to accurately reconstruct the physical proximity relationships between immune and stromal cells and their *in situ* transcriptional responses within the gradient microenvironment *in vitro* or in in vivo models. To fill this cross-scale validation gap, spatial transcriptomics (ST), as a revolutionary multidimensional analytical tool, has been established as the readout method for modern osteoimmunoengineering preclinical evaluation systems. By achieving high-throughput *in situ* capture of the whole transcriptome while fully preserving the tissue’s anatomical topology, ST technology provides a panoramic molecular map for decoding immunometabolic cascades and material intervention dynamics within complex models.

When processing complex hard tissue samples like the TBI, which are rich in dense type I collagen and mineralized matrices and often require decalcification and formalin-fixed paraffin-embedded (FFPE) processing, traditional spatial gene capture faces technical barriers where RNA is highly prone to severe degradation. Cutting-edge spatial transcriptomics platforms based on multiplexed fluorescence *in situ* hybridization and high-resolution imaging have demonstrated outstanding robustness when dealing with such orthopedic preclinical model samples. Following rigorous methodological benchmarking, these imaging platforms can achieve precise transcript quantification at the subcellular level in FFPE sections with extremely high autofluorescence backgrounds, without sacrificing target detection specificity and sensitivity (Wang H. et al., 2025). Relying on these advanced spatial analysis technologies, researchers are able to construct organ-level, high-fidelity, pan-tissue single-cell spatial atlases encompassing fibroblasts, chondrocytes, and monocytes/macrophages across the anatomical scales of macroscopic organoids and large experimental animals. This high-dimensional anatomical atlas not only finely maps the gradient distribution patterns of healthy stromal cells across different mechanical stress loading zones but also profoundly reveals the highly location-specific transcriptional features and differentiation trajectories exhibited by immune resident cells (Restrepo et al., 2026).

When utilizing advanced animal models or organoids to investigate the destructive mechanisms of chronic pathological environments (such as aging and diabetes) on TBI healing, ST technology provides irreplaceable analytical depth for precisely localizing the core driving zones of immune degeneration. By incorporating spatial heterogeneity and autocorrelation analysis algorithms from geographic information science, researchers can redefine the dynamic boundaries between the pathological matrix and nascent granulation tissue within the damaged enthesis with extremely high mathematical precision (Zormpas et al., 2023). Within this high-dimensional spatial framework, through deep deconvolution and joint analysis of ST and conventional scRNA-seq data, the highly specific senescence-associated secretory phenotype (SASP) is precisely anchored to specific microanatomical lesions. Specific aging and metabolic degeneration pathways, such as the upregulation of CDKN1A expression and key nodes like IGFBP7, do not exhibit homogenized expression within the injury interface; instead, they are highly enriched in microvascular collapse zones or tear edges with extreme mechanical stress concentrations. This dual precise targeting at the organelle and spatial levels visually reveals how senescent endothelial cells or fibroblast-like precursor cells directly induce epigenetic alterations and inflammatory locking in adjacent macrophages within specific physical distances through localized toxic paracrine signaling networks (Zhang W. et al., 2026). This leap from “systemic inflammation” to “localized spatial inflammatory niches” not only completely elucidates the microscopic mechanisms of chronic TBI non-healing but also provides the most precise spatial delivery coordinates for the targeted senolytics and engineered exosome interventions discussed earlier.

The most profound impact lies in spatial transcriptomics thoroughly upending the efficacy evaluation paradigm for intelligent biomaterials and targeted nanodelivery carriers in complex models. In traditional preclinical large animal models, evaluating the efficacy of novel osteoimmune implants often relies heavily on endpoint macroscopic histological staining or biomechanical tensile testing, making it extremely difficult to reveal the early spatial molecular dynamics of material interventions in real-time. Conversely, sequencing-based spatial transcriptomics can not only perform unbiased spatial capture of gene expression on tissue sections but also evaluate the actual effective biological resolution and penetration radius of interventions by precisely quantifying the diffusion dynamics of macromolecules within various dense matrices (You et al., 2024). When biomimetic asymmetric Janus scaffolds with spatiotemporal controlled release characteristics, piezoelectric hydrogels, or micro/nanorobots loaded with mitochondrial antioxidants are implanted into damaged TBI models, ST technology can visually generate a “concentric circle-style” gene reprogramming trajectory map centered on the implant directly on the tissue sections. Researchers can clearly observe how, as the biochemical or physical electrical signal gradients released by the intelligent carrier attenuate outward in space, the macrophage population immediately adjacent to the material interface is the first to downregulate pro-inflammatory transcripts and initiate the M2 polarization program. Furthermore, they can see how this pro-regenerative immune signal penetrates the dense matrix like microscopic ripples, ultimately precisely guiding peripheral, distal tendon stem cells to undergo directional gradient differentiation along the topological structure mapped out by the material in physical space. Through this extremely high-resolution closed loop of spatial molecular tracking validation, osteoimmunoengineering can not only confirm the true immune reprogramming radius of its microenvironmental reconstruction strategies within extremely complex living physiological systems with unprecedented precision but also provide the most authoritative core data support for driving the next-generation of personalized, precise orthopedic intelligent regenerative materials across the translational chasm and toward clinical application (Wang H. et al., 2025; You et al., 2024). As osteoimmune interventions become increasingly complex, the difficulty of bridging the gap from *in vitro* validation to clinical translation escalates sharply. As comprehensively compared in Table 4, compared with traditional models characterized by significant species gaps and systemic black boxes, the advanced validation matrix represented by organoids, organ-on-a-chip, and spatial transcriptomics is reshaping the gold standard for the translational evaluation of orthopedic materials with its unprecedented high fidelity and spatiotemporal resolution.

**TABLE 4:** High-resolution mechanistic readout capabilities of conventional vs. advanced preclinical models in TBI osteoimmunology.

Preclinical validation paradigm	Biomimetic dimensions and core features	Deep mechanistic resolution and multi-omics readout capabilities	Core value in the translation of next-generation smart orthopedic materials	References
3D enthesis organoids	Centimeter-scale macroscopic self-assembly; integration with flexible biomimetic robots to actively apply multidimensional mechanical stretching stress to models	Precisely reproduces the YAP/TEAD4 mechanotransduction cascade *in vitro*; detached from systemic interference to nondestructively track the spatial heterogeneous multidirectional differentiation trajectories of single stem cell lineages with high purity	Reveals how mechano-electrical coupled physical matrices unlock stem cell pluripotency; provides a pure validation bed for personalized verification of epigenetic reprogramming therapies like RNA methylation	Qin et al. (2026), Luo P. et al. (2025), Fang et al. (2025), Mouthuy et al. (2022)
Vascularized microfluidic organ-on-a-chip	Features “M-shaped” interlaced boundary compartmentalized physical isolation; achieves closed-loop wall shear stress (WSS) and microcirculatory immune exudation networks	Capable of dynamically decoding pathological phenotypic transitions of cells induced by abnormal mechanical stress *in situ*; high-resolution quantification of endothelial barrier permeability changes and the “chemotaxis-margination-transendothelial” dynamics of circulating monocytes	Enables large-scale, high-throughput screening and validation of the physical penetration efficacy of smart nanodelivery carriers across pathophysiological barriers within extreme microenvironments highly approximating human hemodynamics	Zhao et al. (2026), Su et al. (2024), Khosravi and Radisic (2026), Park et al. (2019)
Spatial transcriptomics	Perfectly preserves precise *in situ* anatomical physical topology; fully compatible with high-autofluorescence decalcified FFPE hard tissue sections typical in orthopedics	Breaks the restriction of single-cell sequencing requiring tissue dissociation to precisely map intercellular physical proximity interactions; quantifies the actual effective physical impact radius of microenvironmental SASP toxic paracrine signaling	Visualizes “concentric circle-style” smart material drug release/degradation dynamics and *in situ* immune reprogramming diffusion trajectories at the molecular level, completely upending traditional endpoint histological evaluation gold standards	Wang H. et al. (2025), Zormpas et al. (2023), Zhang W. et al. (2026), You et al. (2024)
Large animal *in* *vivo* injury models	Features tissue anatomical dimensions, joint kinematic loading, and a full-size closed-loop metabolic-immune central network that most closely resemble humans	Capable of comprehensively evaluating the macroscopic, long-term reversal effects of local micro/nano targeted interventions on systemic neurocognition, motor function, and systemic inflammatory factors	Serves as the ultimate gatekeeper across the translational “valley of death,” truly validating the anti-adhesion, gradient mineralization, and mechanical fatigue tolerance of macroscopic smart biomimetic materials under long-term complex physiological mechanical loads	Sanz-Ros et al. (2022), Hao et al. (2025)

WSS, wall shear stress; FFPE, formalin-fixed paraffin-embedded; SASP, senescence-associated secretory phenotyp.

Furthermore, the latest paradigm in TBI regeneration fundamentally relies on cross-scale research strategies to bridge the formidable gap between macroscopic joint biomechanics and microscopic single-cell fate. For instance, recent advancements integrating macro-scale gait analysis with micro-scale spatial multi-omics have successfully mapped the specific mechanosensitive progenitor niches at the intact enthesis (Zhang L. et al., 2026; Swaro et al., 2025). Building upon this, state-of-the-art intelligent scaffolds are now being designed with cross-scale hierarchical structures, ranging from macroscopic continuous mechanical gradients down to nanoscopic piezoelectric or topographical cues (Pattnaik et al., 2023; Li J. et al., 2023; Shlapakova et al., 2024; Qu et al., 2026). Crucially, executing these cross-scale strategies requires a multidimensional understanding of how bio-interventions alternative tissue architectures. For instance, recent rigorous studies have combined macroscopic tensile evaluations with micro- and nano-structural profiling of decellularized scaffolds (Sun et al., 2025). These cross-scale analyses revealed that subtle nanoscale disruptions, such as altered collagen D-banding periodicity, directly precipitate the compromise of macroscopic energy dissipation and micromechanical integrity under extreme loading (Sun et al., 2025). This cross-scale evaluation system explicitly correlates macroscopic functional recovery with localized molecular events, thereby establishing the ultimate gold standard for evaluating next-generation immunomodulatory biomaterials.

## 6 Future directions and clinical outlook

**FIGURE 6:**
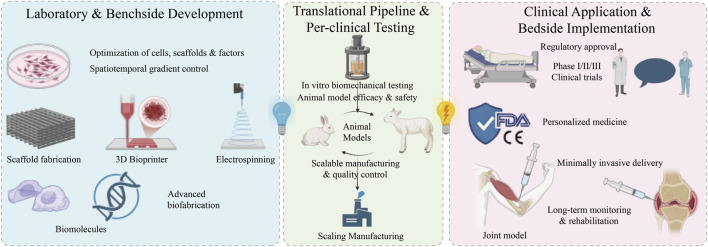
Represents the bench-to-bedside translational continuum for engineered TBI solutions. The strategic pathway advances from fundamental laboratory innovation toward standardized clinical implementation. Key transitional milestones include the utilization of high-fidelity pre-clinical models for mechanistic validation, scalable biomanufacturing, and overcoming regulatory hurdles. The ultimate clinical objective involves integrating artificial intelligence for patient-specific therapeutic design, minimally invasive delivery, and adaptive long-term functional monitoring.

Although osteoimmunoengineering, centered on active immune microenvironment reprogramming, has demonstrated revolutionary potential in TBI regeneration and repair, bridging the gap from basic laboratory proof-of-concept to widespread clinical translation still requires navigating the complex intersections of materials science, systems biology, and translational medicine. As depicted in Figure 6’s translational roadmap from the laboratory to the clinical bedside, future development relies not only on the iteration of the materials themselves but also on the establishment of high-fidelity evaluation systems. To achieve truly scarless anatomical reconstruction in extreme microenvironments accompanied by severe aging and metabolic degenerative diseases, future research paradigms must achieve fundamental underlying breakthroughs in three core dimensions: the deepening of dynamic intelligent response, the spatiotemporal matching of interfacial degradation kinetics, and the digital-intelligent integration of evaluation systems.

### 6.1 Deepening the “closed-loop” of dynamic intelligent response systems

Currently, most immunomodulatory biomaterials applied to TBI repair remain at the primary stage of “open-loop” or pre-programmed release. This unidirectional biochemical or physical intervention often fails to precisely match the highly dynamic and sequential needs of the trauma microenvironment, which fluctuate drastically due to individual patient differences. The evolutionary direction for future intelligent materials must be the construction of “closed-loop” microphysiological systems equipped with autonomous sensing and on-demand feedback capabilities. By deeply integrating bioorthogonal cleavage groups highly sensitive to local tissue markers into macromolecular hydrogel networks, the matrix will be able to act as a real-time sensor-effector for the microenvironment. When an acute inflammatory storm erupts locally or abnormal pathological mechanical stretching occurs, the system can be instantaneously and targetedly degraded or trigger liquid-liquid phase separation, thereby releasing anti-inflammatory exosomes, mitochondria-targeted antioxidants, or specific Piezo1 antagonists in a proportional and step-wise manner; conversely, when inflammation resolves and the tissue enters the remodeling phase, the release behavior will spontaneously cease (Zhai et al., 2026). Furthermore, relying on advanced piezoionics mechanisms, future electroactive implants will no longer be limited merely to providing transient pulsatile electrical signals. Instead, they will be capable of establishing continuous and self-driven ionic currents under long-term physiological loading, providing round-the-clock biochemical homeostasis maintenance for interface tissues deeply trapped in immunometabolic paralysis (Li F. et al., 2026). This leap from passive delivery to active environmental adaptive response will completely eliminate off-target toxicity caused by drug overdosing or targeting failure caused by insufficient release, providing the ultimate materials science solution for personalized osteoimmune intervention.

### 6.2 Spatiotemporal matching of degradation kinetics at heterogeneous interfaces

The asymmetric anatomical structure of the natural TBI dictates a massive time difference in the regeneration rates of its two tissue ends post-injury: the mineralized remodeling of the relatively well-vascularized bone side typically takes months, whereas the matrix reorganization of the avascular dense tendon parenchyma side is even slower. Currently, whether using homogeneous electrospun fibers or hydrogel scaffolds, their global uniform degradation pattern *in vivo* constitutes a fatal hidden danger that induces repair failure. An overly rapid overall degradation causes the immature tendon end to lose its mechanical barrier, inducing catastrophic secondary tearing and fibrotic scar adhesions; conversely, an overly slow degradation triggers a severe stress shielding effect on the bone side, completely blocking the ingrowth and calcium-phosphorus mineralized deposition of osteogenic precursor cells into bone lacunae. Therefore, the research and development of next-generation osteoimmune materials must conquer the engineering barrier of “spatially heterogeneous degradation.” Relying on high-precision multi-material 3D/4D bioprinting technologies and multi-scale hierarchical assembly strategies, future biomimetic scaffolds need to precisely orchestrate gradients of polymer molecular weights, cross-linking densities, and metal-organic frameworks (MOFs) with differential response characteristics within their three-dimensional architectures (Hu et al., 2025; Huang X. et al., 2026). This spatiotemporally controllable gradient degradation system must not only maintain a dynamic balance with the load-bearing capacity of nascent tissues macroscopically at all times but also precisely guide the sequential infiltration and lineage-specific differentiation of macrophages and stem cells at specific anatomical sites as the physical pore size of the matrix evolves spatiotemporally during degradation. Achieving millisecond-level spatiotemporal synchronization between the scaffold degradation rate and the heterogeneous regeneration rhythm of multiple TBI tissues is the core prerequisite for ensuring that nascent gradient fibrocartilage achieves long-term biomechanical stability.

### 6.3 Standardization of preclinical testing platforms and deep integration of “AI-microphysiological systems”

While breaking through material design bottlenecks, basic research and clinical trials must be organically integrated by restructuring evaluation systems. Existing rodent models present non-negligible species differences from humans in terms of skeletal mechanical loading mechanisms, immunosenescence dynamics, and subchondral bone microcirculatory networks. To accelerate the clinical translation of osteoimmunoengineering products, the establishment of standardized validation protocols for macroscopic three-dimensional enthesis organoids and microfluidic organ-on-a-chip platforms is urgently needed (Liu et al., 2025a; Leung et al., 2022). In the future, these highly biomimetic humanized microphysiological systems must serve as mandatory translational validation steps before new materials progress into large experimental animals. Even more profoundly, the comprehensive involvement of artificial intelligence (AI) and machine learning algorithms will completely reshape the digital-intelligent landscape of preclinical validation. Faced with massive fluid dynamics data generated by advanced organ-on-a-chip platforms, real-time electrophysiological pulses monitored by microelectrode arrays, and the *in situ* sequencing data from ultra-high-resolution spatial transcriptomics detailed earlier, traditional biostatistical analysis has already reached its limits. By training deep graph neural networks and generative AI large models, researchers can decode spatial proximity communication among immune cells with high precision amidst complex omics noise, and even predict in advance the penetration trajectories and degradation kinetics of specific intelligent micro/nanorobots within extremely dense scar networks (Cao et al., 2025; Zormpas et al., 2023). This entirely new closed-loop ecosystem of “AI-assisted design—high-throughput organoid screening—precise large animal validation” will not only drastically shorten the research and development cycle of novel materials targeting chronic osteoimmune diseases but also signifies that regenerative medicine for the TBI is unstoppably marching into a brand new era of digitalization, precision, and personalization.

## 7 Conclusion

The successful regeneration of the tendon-bone interface (TBI) under chronic degenerative conditions demands a fundamental paradigm shift from passive structural scaffolding to active microenvironment reprogramming. As elucidated in this review, chronic pathologies such as ageing, diabetes, and rheumatoid arthritis drive a catastrophic collapse of the local immune-metabolic network, trapping resident cells in profound states of senescence, metabolic paralysis, and inflammatory locking. To overcome these hierarchical barriers, modern osteoimmunoengineering seamlessly integrates targeted biochemical cues with dynamic biophysical stimuli to achieve precise spatiotemporal immunomodulation.

Furthermore, transitioning from traditional flat cultures to highly biomimetic preclinical models, including 3D enthesis organoids and multi-chamber microfluidic chips is strictly necessitated to bridge the formidable translational gap. Ultimately, the future of TBI regeneration lies in the convergence of closed-loop smart biomaterials, advanced spatial multi-omics, and artificial intelligence. By fundamentally decoding and therapeutically rewriting the pathological cross-talk at the enthesis, these interdisciplinary strategies hold the profound potential to finally translate precision orthopaedic regenerative medicine from the laboratory to the clinic.
