# Supplementary material for: Immunoengineering in the field of tendon and bone regeneration: immunomodulatory biomaterials, delivery platforms, and preclinical models for chronic diseases
Source: Front Bioeng Biotechnol. 2026 May 21;14:1844904. doi: 10.3389/fbioe.2026.1844904 (PMC13233466; doi:10.3389/fbioe.2026.1844904)
Supplement: Supplementary file 1 [file Table1.docx]

**Table 1. In-depth mechanistic summary of immunometabolic and mechanotransductive collapse at the TBI under chronic degenerative conditions.**

| Pathological Trigger | Core Cellular and Subcellular Targets | Deep Molecular and Epigenetic Mechanisms | Immune-Metabolic-Mechanical Cascade Collapse Outcomes | References |
| --- | --- | --- | --- | --- |
| Inflammaging | Macrophages, tendon stem/progenitor cells (TSPCs), and local microvascular endothelial cells | Abnormal locking of NF-κB/MAPK pathways; DNA damage response (DDR) triggered by telomere attrition; profound epigenetic silencing of stem cells due to abnormal accumulation of repressive transcriptional marks. | Toxic paracrine diffusion of senescence-associated secretory phenotype (SASP); irreversible M1 polarization locking of macrophages; comprehensive stagnation of tissue-level collagen anabolism and remodeling failure. | (3,57,58) |
| Diabetes / Severe Metabolic Disorders | Mitochondrial network, subchondral osteoblasts | Severe damage to the electron transport chain (ETC) and mtROS burst; imbalanced rewiring of glycolytic metabolism; non-enzymatic pathological cross-linking induced by advanced glycation end products (AGEs). | Collapse of mitochondrial bioenergetics, inducing cellular ferroptosis/apoptosis; profound microenvironmental hypoxia; significantly increased matrix brittleness, accompanied by fatal heterotopic ossification. | (59–61) |
| Abnormal Mechanical Stress & Neural Degeneration | Interfacial fibroblasts, peripheral sensory neuron endings | Overactivation of the Piezo1-YAP mechanotransduction axis induces destructive inward calcium transients; exhaustion of the synthesis and release pools for sensory neurotransmitters (e.g., CGRP, NPY). | Catastrophic conversion of cellular contractile phenotype to pro-inflammatory/pro-fibrotic synthetic phenotype; loss of immune privilege due to severed "neural-immune" communication; progressive mechanical tearing of the interfacial microstructure. | (62–64) |

*Abbreviations: DDR, DNA damage response; SASP, senescence-associated secretory phenotype; mtROS, mitochondrial reactive oxygen species; ETC, electron transport chain; NPY, neuropeptide Y.*

## Table 2. Summary of core immune-regulatory mechanisms at the TBI and corresponding engineered intervention strategies.

| Core Regulatory Axis | Key Cellular Targets | Physiological Role in Native Healing | Pathological Disruption in Chronic TBI | Advanced Immunoengineering Interventions | References |
| --- | --- | --- | --- | --- | --- |
| Macrophage Polarization Dynamics | M1/M2 Macrophages, Fibroblasts, Osteoprogenitors | Transition to the M2 phenotype initiates TGF-β/VEGF-driven collagen synthesis, microvascular ingrowth, and robust tissue remodeling. | Epigenetic "M1 locking" via NF-κB/MAPK hyperactivation; continuous ROS burst completely abrogates the transition to the proliferative phase. | Antioxidant nanozymes and specific nanotopographical cues to clear ROS, activate PI3K/AKT survival pathways, and forcibly drive M2 conversion. | (23,28,30) |
| Piezo1-YAP Mechanotransduction | Interfacial Fibroblasts, Periosteal Progenitor Cells | Translates physical stress into YAP nuclear translocation to precisely regulate the osteogenic vs. tenogenic lineage plasticity of stem cells. | Abnormal mechanical stress triggers destructive inward calcium transients, inducing malignant macrophage-fibroblast crosstalk and hypertrophic scarring. | Spatiotemporal delivery of Piezo1 antagonists via smart hydrogels to decouple aberrant mechanotransduction and prevent heterotopic ossification. | (42,45,48) |
| Neuro-Immune-Skeletal Axis | Sensory Nerve Endings, Neutrophils, MSCs | Neuropeptides (e.g., CGRP) rapidly resolve inflammation (efferocytosis) and perfectly couple immune resolution with matrix deposition. | Age/diabetes-induced sensory denervation halts Sema3A/CGRP release, severely depriving the local stem cell pool of critical neurodifferentiation signals. | Electroactive scaffolds and PEMFs to reawaken residual neurons; phase-separation hydrogels for the biomimetic on-demand release of sensory neurotransmitters. | (53,56,59) |

**Notes:** This table synthesizes the physiological roles and pathological disruptions of three core regulatory axes and their respective bioengineering intervention strategies as discussed in Section 3.

**Table 3. Mechanistic classification of advanced osteoimmunomodulatory biomaterials and spatial delivery platforms for deep-tissue reprogramming**

| Engineered Intervention Chassis/Carrier | Targeted Biochemical Signals/Physical Cues | Master Molecules and Deep Transduction Pathways | Tissue-Level Spatiotemporal Immune Reprogramming Effects | References |
| --- | --- | --- | --- | --- |
| Dynamic Piezoelectric Bioelectronic Interfaces | In situ ultrasound/mechano-electrical conversion microelectric fields; Neuropeptide molecules released via liquid-liquid phase separation (LLPS) | Specifically activates PI3K/AKT and FAK/AKT pro-survival pathways; targetedly upregulates PIEZO1/2 ion channels in stromal cells; promotes MFN/OPA1-mediated mitochondrial network fusion. | Step-wise ROS clearance and potent M2 macrophage reprogramming; electrical stimulation triggers sustained, on-demand release of peptide condensates, precisely reconstructing the microenvironmental "neural-immune-skeletal" communication axis. | (63,64,84) |
| Janus Asymmetric Microfluidic Membranes | Gradient metal ions (Ca/P vs. Mg/Zn); Electric double layer (EDL) dynamic ionic currents | Relying on piezoionics mechanisms, drives asymmetric migration of anions and cations via localized applied voltage or extremely weak mechanical forces, achieving artificial synapse-level unidirectional ionic rectification. | Macroscopic asymmetric biomimetic topology completely blocks exogenous fibrotic scar adhesions; microscopic programmable ionic gradients guide spatial compartmentalized polarization of macrophages and drive gradient mineralization of the natural tendon-to-bone interface. | (90,92,93) |
| Biological Micro/Nanorobots & Subcellular Targeted Platforms | Lipid peroxidation inducers; Dodecyltriphenylphosphonium (dTPP)-MitoQ | Targets unique vulnerabilities of senescent cells, hijacking ALOX15 lipoxygenase to trigger specific ferroptosis; penetrates the bilayer membrane to activate the Nrf2/ARE antioxidant response element master pathway in the nucleus. | Relies on the "enzyme-macrophage switching (EMS)" mechanism to penetrate dense scars and target extremely deep lesions; forcibly downregulates mitochondrial oxygen consumption and induces adaptive glycolytic metabolic transitions in immune cells. | (59,94–96) |
| Engineered Stem Cell Exosomes & Living Cell Therapies | Immune licensing (IFN-γ preconditioning) enriched specific miRNAs/Hsp70; uPAR-targeted receptors | Targetedly activates SIRT1/TIMP1/Notch1 tissue homeostasis maintenance hubs; triggers ALKBH5-dependent m6A RNA demethylation modifications; CAR-T cell-mediated long-acting immune surveillance. | Potently reverses the transcriptional profile of senescent cells without altering the genome; assists in folding damaged proteins, suppressing macrophage inflammatory storms within an extremely short time window. | (99,103,107,111) |

*Abbreviations: LLPS, liquid-liquid phase separation; EDL, electric double layer; EMS, enzyme-macrophage switching; ARE, antioxidant response element*

**Table 4. High-resolution mechanistic readout capabilities of conventional vs. advanced preclinical models in TBI osteoimmunology**

| Preclinical Validation Paradigm | Biomimetic Dimensions and Core Features | Deep Mechanistic Resolution and Multi-Omics Readout Capabilities | Core Value in the Translation of Next-Generation Smart Orthopedic Materials | References |
| --- | --- | --- | --- | --- |
| 3D Enthesis Organoids | Centimeter-scale macroscopic self-assembly; integration with flexible biomimetic robots to actively apply multidimensional mechanical stretching stress to models. | Precisely reproduces the YAP/TEAD4 mechanotransduction cascade in vitro; detached from systemic interference to nondestructively track the spatial heterogeneous multidirectional differentiation trajectories of single stem cell lineages with high purity. | Reveals how mechano-electrical coupled physical matrices unlock stem cell pluripotency; provides a pure validation bed for personalized verification of epigenetic reprogramming therapies like RNA methylation. | (111,114–116) |
| Vascularized Microfluidic Organ-on-a-Chip | Features "M-shaped" interlaced boundary compartmentalized physical isolation; achieves closed-loop wall shear stress (WSS) and microcirculatory immune exudation networks. | Capable of dynamically decoding pathological phenotypic transitions of cells induced by abnormal mechanical stress in situ; high-resolution quantification of endothelial barrier permeability changes and the "chemotaxis-margination-transendothelial" dynamics of circulating monocytes. | Enables large-scale, high-throughput screening and validation of the physical penetration efficacy of smart nanodelivery carriers across pathophysiological barriers within extreme microenvironments highly approximating human hemodynamics. | (62,117–119) |
| Spatial Transcriptomics | Perfectly preserves precise in situ anatomical physical topology; fully compatible with high-autofluorescence decalcified FFPE hard tissue sections typical in orthopedics. | Breaks the restriction of single-cell sequencing requiring tissue dissociation to precisely map intercellular physical proximity interactions; quantifies the actual effective physical impact radius of microenvironmental SASP toxic paracrine signaling. | Visualizes "concentric circle-style" smart material drug release/degradation dynamics and in situ immune reprogramming diffusion trajectories at the molecular level, completely upending traditional endpoint histological evaluation gold standards. | (122,124–126) |
| Large Animal In Vivo Injury Models | Features tissue anatomical dimensions, joint kinematic loading, and a full-size closed-loop metabolic-immune central network that most closely resemble humans. | Capable of comprehensively evaluating the macroscopic, long-term reversal effects of local micro/nano targeted interventions on systemic neurocognition, motor function, and systemic inflammatory factors. | Serves as the ultimate gatekeeper across the translational "valley of death," truly validating the anti-adhesion, gradient mineralization, and mechanical fatigue tolerance of macroscopic smart biomimetic materials under long-term complex physiological mechanical loads. | (104,106) |

*Abbreviations: WSS, wall shear stress; FFPE, formalin-fixed paraffin-embedded; SASP, senescence-associated secretory phenotyp*
